# Intermediate Conductance Calcium‐Dependent Potassium Channel (K_Ca_3.1) Interacting Proteins Using Turboid‐Based Proximity Labeling Technology: Insights Into Interactome and Related Signaling Pathways in Pancreatic Tumors

**DOI:** 10.1002/jcp.70092

**Published:** 2025-09-15

**Authors:** Veronica Carpanese, Soha Sadeghi, Luca Matteo Todesca, Ildikò Szabò, Vanessa Checchetto

**Affiliations:** ^1^ Department of Biology University of Padova Padova Italy

**Keywords:** cell signaling pathways, interactome analysis, K_Ca_3.1 ion channel, pancreatic tumors, proximity proteomics

## Abstract

K_Ca_3.1 ion channel is a calcium‐activated potassium channel expressed in various tissues, showing dual localization to the plasma membrane and to mitochondria. This channel is highly expressed in numerous cancers and has been implicated in the regulation of proliferation and migration. The molecular details of the signaling pathways linked to regulation exerted by K_Ca_3.1 in cancer cells are, however, not fully elucidated yet. Therefore, we determined the interactome of K_Ca_3.1 using proximity labeling in intact KPC pancreatic cancer cells that mirror the aggressive metastatic behavior of human pancreatic cancer. The results highlight several novel interactors, including those residing in intracellular membranes. The K_Ca_3.1 channel proxisome and related pathways are discussed in light of our current knowledge about K_Ca_3.1 and pancreatic cancer, available in public databases.

## 1 Introduction

The *KCNN4* gene encodes a calcium‐activated voltage‐independent potassium channel K_Ca_3.1 (also called IK1 or SK4 or Gardos channel) with conductance values ranging up to 90 pS in 150 mM KCl. This channel has been observed to function as a heterotetramer and is activated by intracellular calcium at sub‐micromolar concentrations, leading to membrane hyperpolarization. This, in turn, facilitates the activation of transient receptor potential vanilloid (TRPV) and calcium release activated calcium current (CRAC) channels and enhances calcium influx through the plasma membrane in several cell types, including cancer cells and immune cells, where K_Ca_3.1 is expressed (Feske et al. 2019, 2015).

K_Ca_3.1 is present and functional also in the inner mitochondrial membrane, as observed by Western blot and patch clamping of mitoplasts (inner membrane vesicles deprived of the outer membrane) (De Marchi et al. 2009; Sassi et al. 2010; Kovalenko et al. 2016; Bulk et al. 2022; Szabo and Szewczyk 2023; Todesca et al. 2024). The mitochondrial channel displays the typical biophysical and pharmacological features of the plasma membrane located K_Ca_3.1, suggesting that similarly to the K_v_1.3 channel (Capera et al. 2022, 2021), the same gene encodes the two isoforms (Szabo and Zoratti 2014). K_Ca_3.1 contributes to potassium flux across the mitochondrial inner membrane, helping to stabilize the mitochondrial membrane potential (Δ_Ψm_), which is crucial for regulating several mitochondrial processes, therefore supporting pancreatic ductal adenocarcinoma (PDAC) growth (Hashimoto et al. 2022, Decker and Funai 2024). The regulation of Δ_Ψm_ affects mitochondrial bioenergetic and ATP production, being a possible support in the high energy demand of PDAC cells (Hashimoto et al. 2022; Huang et al. 2021). Moreover, PDAC cells rely on metabolic flexibility, and mitochondrial K_Ca_3.1 may facilitate adaptive metabolic shifts (Yamamoto et al. 2022): by supporting oxidative phosphorylation (OXPHOS) and mitochondrial efficiency, K_Ca_3.1 can help PDAC cells to survive in a hypoxic tumor microenvironment (Kovalenko et al. 2016; Sadozai et al. 2024). Lastly, K_Ca_3.1‐mediated regulation of mitochondrial ion homeostasis influences ROS production, a process implicated in tumor progression and chemoresistance (Moloney and Cotter 2018; Qin et al. 2023) and may enhance survival signaling in PDAC cells (Cheung et al. 2020). Given its extensive role in the mitochondria, K_Ca_3.1 could be a potential target to modulate mitochondrial homeostasis in PDAC. Indeed, pharmacological inhibition or silencing of K_Ca_3.1 modulated oxidative phosphorylation (Kovalenko et al. 2016) and a mitochondriotropic version of the small molecule inhibitor TRAM‐34 (mitoTRAM‐34) efficiently killed tumor cells by inducing apoptosis (Bachmann et al. 2022). Interestingly, sub‐lethal concentrations of this drug reduced melanoma migration and metastasis in vivo via a ROS‐BNIP3‐Rho‐GTPase pathway (Bachmann et al. 2022). Altogether, these findings highlight both the mitochondrial and plasma membrane‐located K_Ca_3.1 as a potential therapeutic target against various types of cancer, including the PDAC.

Indeed, K_Ca_3.1 expression is upregulated in a variety of tumor tissues (Quast et al. 2012; Kovalenko et al. 2016; Mohr et al. 2019; Wen et al. 2020; Bulk et al. 2022; Chen et al. 2022; Faouzi et al. 2016), including pancreatic ductal adenocarcinoma (Soret et al. 2023), where the channel expression was proposed to represent a prognostic biomarker (Jiang et al. 2017). K_Ca_3.1 drives the progression and metastasis of PDAC via the MET‐mediated AKT signaling pathway (Mo et al. 2022). Conversely, knockdown or pharmacological modulation of the channel led to apoptosis (Bachmann et al. 2022) and cell cycle arrest in PDAC cells (Mo et al. 2022). K_Ca_3.1 has also been linked to PDAC and stellate cell migration (Bonito et al. 2016; Storck et al. 2017; Guéguinou et al. 2015).

PDAC is among the most lethal solid tumors, characterized by an extremely poor prognosis, with a 5‐year survival rate of less than 6% (Halbrook et al. 2023). The deep anatomical location of the pancreas presents a significant challenge to routine screening, resulting in late‐stage diagnoses in more than half of PDAC cases, and consequently, surgical resection is no longer a possible option (Jiang and Sohal 2023). Chemotherapy remains one of the few treatment options available, but it is hindered by drug resistance, which is a major challenge in improving overall survival rates (Jiang and Sohal 2023). Despite extensive research efforts, no highly effective therapeutic strategies have been developed. Therefore, further exploration of the molecular mechanisms driving PDAC is essential to identify novel therapeutic targets and improve treatment outcomes. As mentioned above, K_Ca_3.1 might represent a promising target, but more information is needed about its function in PDAC cells/tissues, as its molecular role in PDAC progression remains largely unknown. Furthermore, recent data suggest that some of the known pharmacological modulators of this channel may act independently of K_Ca_3.1 expression in the plasma membrane (Zuccolini et al. 2023), calling for caution when interpreting results using these drugs.

To gain more information about K_Ca_3.1, in this study, we used Turbo‐proximity‐dependent BioID (Roux et al. 2013, 2018, 2012; Cho et al. 2020) to identify and characterize components of the K_Ca_3.1 signalling pathways. Recently, proximity labelling techniques (BioID; TurboID and Apex) have emerged as powerful tools for capturing ion channel interactions in living cells (Park et al. 2020; Redel‐Traub et al. 2021; Bowen et al. 2024; Carpanese et al. 2024; Gallo et al. 2024; Krajewska et al. 2024; Prosdocimi et al. 2024; Kour et al. 2025). These techniques overcome the limitations imposed by immune‐precipitation and mass spectrometry (Sears et al. 2019). Importantly, in all reported cases so far, the fusion of the biotinylating enzyme to different ion channels did not significantly alter their function or subcellular localization to the plasma membrane or to mitochondria (Krajewska et al. 2024).

Studying the protein interactome of K_Ca_3.1 is crucial for uncovering its novel functions. Interactome analysis can reveal previously unidentified binding partners, providing new insight into how K_Ca_3.1 contributes to tumor growth, invasion, and metastasis. Additionally, identifying its regulatory mechanisms at the protein level can clarify the signaling pathways and cellular processes influenced by its activity, which is essential for developing targeted therapeutic strategies. Traditional methods such as immunoprecipitation often struggle to detect weak or transient protein interactions, particularly those involved in dynamic cellular responses, but advanced interactome analysis can overcome these limitations, offering a more comprehensive understanding of K_Ca_3.1 role. Furthermore, if this channel were found to interact with key oncogenic or tumor‐suppressive proteins, this interaction could provide a potential drug target. By mapping the K_Ca_3.1 interactome, here we identified key molecular targets that may serve as valuable candidates for future mono‐ and combinatorial therapeutic strategies in pancreatic cancer.

## 2 Materials and Methods

### 2.1 Cell Culture

KPCY murine Pancreatic Ductal Adenocarcinoma (PDAC) cells (kindly gifted by A. Carrer), HEKT, and HEK293 were cultured in DMEM (4.5 g/L d‐glucose) supplemented with 10% (v/v) Fetal Bovine Serum (FBS), 100 U/mL penicillin G, and 0.1 mg/mL streptomycin. Cells were maintained in a humidified incubator at 37°C and 5% CO2, passaged twice a week when confluent. All material for cell culture was purchased from Gibco.

### 2.2 Generation of the Lentiviral‐Inducible Plasmid Coding for K_Ca_3.1‐FLAG‐TurboID Protein

The murine *KCNN4* coding sequence was amplified from the *KCNN4*‐Myc‐DDK plasmid (Origene Cat. No. MR206777) with Phusion DNA Polymerase (England Biolabs Cat. No. M0530S) using primers harboring attB recombination sites (Primer FOR: 5′‐GGGGACAAGTTTGTACAAAAAAGCAGGCTTCACC ATGGGCGGGGAGCTGGTGA‐3′; Primer REV: 5′‐ GGGGACCACTTTGTACAAGAAAGCTGGGTG TGTGGCCTCCTGGCTGGGTTC). Following Gateway Cloning protocol (Katzen 2007) the PCR product was cloned into pDONR221 Vector (ThermoFischer Scientific Cat. No. 12536017) to create an Entry Clone plasmid containing *KCNN4* coding sequence flanked by attL recombination sites. The Entry Clone vector and the Destination Vector (Addgene plasmid #194073) containing FLAG‐TurboID coding sequence were then used to generate the lentiviral Expression Clone vector coding for K_Ca_3.1‐FLAG‐TurboID protein under the control of the TRE‐inducible promoter.

### 2.3 Generation of Stable Cell Lines via Lentiviral Transduction

To generate KPCY cell lines stably expressing K_Ca_3.1‐FLAG‐TurboID or FLAG‐TurboID, 1,000,000 HEK293T cells were seeded in 10 mm^2^ culture dishes in standard culture medium. Two days after the seeding, they were transfected with psPAX2 plasmid (Addgene plasmid #12260), VSV.G (Addgene plasmid #14888), and the Expression Clone Vector coding for K_Ca_3.1‐FLAG‐TurboID protein or the plasmid coding for FLAG‐TurboID protein using polyethyleneimine 1 mg/mL (Merck Cat. No. 919012) to generate lentiviral particles. After 48 h hours, the medium containing lentiviruses was collected, filtered with 0.45 μM‐pore filters, and used to transducing KPCY cells. The transduced cells were then selected with 30 μg/mL of blasticidin (InvivoGen Cat. No. ant‐bl‐05) for 96 h to obtain stable cell lines. The genomic insertion of *KCNN4*‐FLAG‐TurboID sequence or FLAG‐TurboID sequence was checked in the blasticidin‐resistant cell lines by PCR. To generate the cell lines overexpressing K_Ca_3.1‐GFP‐myc, HEK293 and KPCY cells were infected with a lentiviral plasmid encoding for K_Ca_3.1‐GFP‐myc and selected using puromycin (InvivoGen Cat. No. ant‐pr‐1).

### 2.4 Biotinylation Analysis and Western Blot Analysis

100,000 KPCY cells expressing the fusion protein K_Ca_3.1‐FLAG‐TurboID and FLAG‐TurboID (cell line used as control in TurboID experiments) were seeded in 6‐w plate in standard culture medium. The day after both the cell lines were cultured for 24 h in medium supplemented with 1 μg/mL doxycycline (Merck Cat. No. D9891) to promote the expression of either K_Ca_3.1‐FLAG‐TurboID or FLAG‐TurboID proteins. Afterward, the biotinylation was induced by administering to the cells medium containing both 1 μg/mL doxycycline and 50 μM biotin for 1, 3, and 5 h. To assess the level of biotinylation, cells were washed twice with PBS and resuspended in RIPA lysis buffer (25 mM Tris HCl pH 7.6, 150 mM NaCl, 1% NP‐40, 1% sodium deoxycholate, 0.1% EDTA) supplemented with protease inhibitor cocktail (Merck Cat. No. P2714). Lysates were incubated on ice for 30 min, then centrifuged at 4°C, 20,000 g for 10 min. The resulting supernatants were collected, proteins quantified by BCA assay, dissolved in loading buffer (30% glycerol, 0.313 M Tris‐627 HCl (pH 6.8), 9% SDS, 0.1 M DTT, 0,1% blue bromophenol), boiled at 95°C for 10 min and then used for immunoblotting analyses. For biotinylation experiments, 50 µg of proteins were separated on 4%–12% on Bis‐Tris gels (Genscript Cat. No. M00652) and then blotted at 4°C for 1 h 15 min at 300 mA to nitrocellulose membranes (Amersham Cat. No. GE10600002). Membranes were blocked for 30 min in 1% Bovine Serum Albumin (BSA) in PBS with 0.2% Triton X‐100 at room temperature (RT) and incubated for 40 min in the same buffer with horse radish peroxidase (HRP)‐conjugated streptavidin (Thermo Fisher Scientific Cat. No #200‐403‐095; 1:40,000). After three washes with PBS, membranes were incubated in a luminol and peroxide solution (in ratio 1:1) (Bio‐Rad) for 5 min and the chemiluminescent signals detected using the ChemiDoc Gel Imaging System (BioRad). For classical Western blot analysis analysis, 50 proteins were separated on 4%–12% Bis‐Tris gels and then transferred to nitrocellulose membranes as described above. Membranes were blocked in 5% milk (Merck Cat. No. 70166) in Tris‐Buffered Saline (TBS) for 1 h at RT and incubated overnight at 4°C with the primary antibody anti‐FLAG (1:1000 in TBS; Merck F7425). Anti‐rabbit (1:5000 in TBS‐Tween20 0.05%) HRP‐conjugated secondary antibodies were used for 1 h at RT in TTBS.

### 2.5 BioId Pulldown

10^6^ KPC cells expressing the fusion protein K_Ca_3.1‐FLAG‐TurboID and the corresponding control cells expressing FLAG‐TurboID, were seeded into two 10 mm^2^ culture dishes per condition. To promote the expression of either K_Ca_3.1‐FLAG‐TurboID or FLAG‐TurboID proteins, the day after the seeding, both cell lines were cultured for 24 h in medium supplemented with 1 μg/mL doxycycline. Afterward, the biotinylation was induced by treating the cells for 3 h with medium containing both 1 μg/mL doxycycline and 50 μM biotin (Merck Cat. No. B4501). Cells were then washed with PBS, collected, and lysed with RIPA lysis buffer supplemented with protease and maintained on ice for 15 min. Cells were collected by centrifugation for 10 min at 13,000*g*, 4°C, and the supernatant was used for Streptavidin‐based pull‐down using MyOne Dynabeads Streptavidin C1 (Thermo Fisher Scientific Cat. No. 65002) as described previously (Le Sage et al. 2016). Western blot or mass spectroscopy was performed to identify biotinylated proteins. The whole experiment was repeated three independent times.

### 2.6 Mass Spectrometry

#### 2.6.1 Sample Preparation SP3 and TMT Labeling, Oasis

The reduction of disulfide bridges in cysteine‐containing proteins was performed with DTT (56°C, 30 min, 10 mM in 50 mM Hepes, pH 8.5). Reduced cysteines were alkylated with 2‐chloroacetamide (room temperature, in the dark, 30 min, 20 mM in 50 mM Hepes, pH 8.5). Samples were prepared using the SP3 protocol (Hughes et al. 2014), and trypsin (sequencing grade, Promega) was added in an enzyme‐to‐protein ratio of 1:50 for overnight digestion at 37°C. The peptides were labeled with TMT11plex (Werner et al. 2014) Isobaric Label Reagent (Thermo Fisher Scientific) according to the manufacturer's instructions. Samples were combined for the TMT11plex, and for further sample cleanup, an OASIS HLB μElution Plate (Waters) was used. Offline high‐pH reverse‐phase fractionation was carried out on an Agilent 1200 Infinity high‐performance liquid chromatography system, equipped with a Gemini C18 column (3 μm, 110Å, 100 × 1.0 mm, Phenomenex).

#### 2.6.2 LC‐MS/MS Acquisition

An UltiMate 3000 RSLC nano‐LC system (Dionex) fitted with a trapping cartridge (μ‐Precolumn C18 PepMap 100, 5 μm, 300 μm i.d. × 5 mm, 100 Å) and an analytical column (nanoEase M/Z HSS T3 column 75 μm × 250 mm C18, 1.8 μm, 100Å, Waters) were used. The trapping was carried out with a constant flow of trapping solution (0.05% trifluoroacetic acid in water) at 30 μL/min onto the trapping column for 6 min. Subsequently, the peptides were eluted via analytical column running solvent A (0.1% formic acid in water and 3% dimethyl sulfoxide (DMSO)) with a constant flow of 0.3 μL/min, with an increasing percentage of solvent B (0.1% formic acid in acetonitrile, 3% DMSO). The outlet of the analytical column was coupled directly to an Orbitrap Fusion Lumos Tribrid Mass Spectrometer (Thermo) using the Nanospray Flex ion source in positive ion mode.

The peptides were introduced into the Fusion Lumos via a Pico‐Tip Emitter 360 μm OD × 20 μm ID, 10 μm tip (New Objective), and an applied spray voltage of 2.4 kV. The capillary temperature was set at 275°C. The full mass scan was acquired with mass ranges of 375–1500 mass/charge ratio (m/z) in profile mode in the orbitrap with a resolution of 120,000. The filling time was set at a maximum of 50 ms with a limitation of 4 × 10^5 ^ions. Data‐dependent acquisition was performed with the resolution of the Orbitrap set to 30,000, with a fill time of 94 ms and a limitation of 1 × 10^5 ^ions. A normalized collision energy of 38 was applied. The MS2 data were acquired in profile mode.

#### 2.6.3 MS Data Analysis—IsobarQuant

IsobarQuant and Mascot (v2.2.07) were used to process the acquired data, which were searched against a UniProt *Mus musculus* proteome database (UP000000589) containing common contaminants and reversed sequences. The following modifications were included in the search parameters: carbamidomethyl (C) and TMT11 (K) (fixed modification), acetyl (protein N‐terminal), oxidation (M), and TMT11 (N‐terminal) (variable modifications). For the full scan (MS1), a mass error tolerance of 10 parts per million was set, and for an MS/MS (MS2) spectra of 0.02 Da. Further parameters were established: trypsin as protease with an allowance of a maximum of two missed cleavages: a minimum peptide length of seven amino acids; at least two unique peptides were required for protein identification. The FDR at the peptide and protein level was set to 0.01.

### 2.7 Mass Spectrometry Data Analysis

The raw IsobarQuant output files (protein.txt–files) were processed using the R programming language (http://www.r-project.org). Only proteins that were quantified with at least two unique peptides and identified in all mass spec runs were considered for analysis. Raw reporter ion intensities (signal_sum columns) were first cleaned for batch effects using limma (Ritchie et al. 2015) and further normalized using vsn (variance stabilization normalization) (Huber et al. 2002). Missing values were imputed with the “knn” method using the Msnbase package (Gatto and Lilley 2012). The differential expression of the proteins was tested using the limma package. The replicated information was added as a factor in the design matrix given as an argument for the limma lmFit function. Furthermore, the imputed values were given a weight of 0.05 in the “lmFit” function. A protein was annotated as a hit with an FDR smaller than 5% and a fold change of at least 100% (1.5‐fold change).

### 2.8 Immunoprecipitation

Cells stably expressing K_Ca_3.1‐GFP‐myc and corresponding control cells were lysed in NP‐40 lysis buffer (50 mM Tris pH 7.4, 250 mM NaCl, 5 mM EDTA, 50 mM NaF, 1 mM Na_3_VO_4_, 1% NP40, 0.02% NaN_3_) supplemented with protease inhibitors, followed by centrifugation at 15,000*g* for 15 min at 4°C. The resulting supernatant was incubated overnight at 4°C with Dynabeads Protein G (Thermo Fisher Scientific) conjugated to either anti‐GFP or anti‐myc antibodies under gentle rotation. The following day, beads were washed three times with cold PBS. Bound proteins were eluted in 30 μL of elution buffer, denatured with SDS sample buffer, and boiled for 10 min at 95°C before SDS‐PAGE and Western blot analysis. For store‐operated calcium entry (SOCE) activation experiments, cells were first treated with 2 μM thapsigargin in Ca²⁺‐free HBSS supplemented with 500 μM EGTA and 0.5 mM MgCl₂ for 10 min to deplete intracellular calcium stores. Cells were then incubated in HBSS containing Ca²⁺ for an additional 10 min to activate SOCE. After treatment, cells were harvested and lysed in NP‐40 lysis buffer supplemented with protease inhibitors for immunoprecipitation analysis. For the detection of STIM1 and KCa3.1‐GFP, membranes were simultaneously incubated with primary antibodies against GFP (1:1000) and STIM1 (1:1000) overnight at 4°C. The following day, membranes were washed and incubated simultaneously with secondary antibodies: anti‐mouse for GFP detection and anti‐rabbit for STIM1 detection.

### 2.9 Confocal Microscopy

For immunofluorescence analysis, KPCY or HEK293 cells were seeded on 12 mm glass coverslips in 12‐well plates to reach approximately 60% confluence the following day. Cells were washed once with PBS, fixed in 3.8% PFA for 15 min, washed three times with PBS. Permeabilization was performed using 0.1% Triton X‐100 (Sigma‐Aldrich) in PBS for 10 min, followed by three PBS washes. Cells were then blocked for 1 h at room temperature in PBS with 1% BSA and 5% goat serum and incubated overnight at 4°C with anti‐TOMM20 antibody (1:400, GeneTex, GTX133756). The next day, cells were washed three times in PBS and incubated for 1 h at room temperature with Alexa Fluor 568‐conjugated anti‐rabbit secondary antibody (1:1000, Thermo Fisher Scientific, #A‐11011) or with 150 nM Alexa Fluor 568‐conjugated Phalloidin (Thermo Fisher Scientific, #A12381) in 5% goat serum in PBS. Samples were washed three times in PBS and mounted with ProLong Gold Antifade Mountant with DAPI (Thermo Fisher Scientific), and images were acquired with a Zeiss LSM900 Airyscan2 confocal microscope. For mitochondria labeling with MitotrackerRed(Thermo Fisher Scientific) staining cells were wash twice with PBS and then incubated with 100 nM MitotrackerRed at 37°C for 30 min. Nuclei were subsequently stained with Hoechst dye for 10 min at room temperature. After staining, cells were washed 1x in PBS, fixed in 3.8% PFA for 15 min mounted with ProLong Gold Antifade Mountant with DAPI (Thermo Fisher Scientific), and images were acquired with a Zeiss LSM900 Airyscan2 confocal microscope.

### 2.10 Gene Ontology and KEGG Analysis

The list of the selected proteins was used to identify significantly enriched functional categories. Enrichment analyses were performed using the clusterProfiler R package (Wu et al. 2021) on GO categories of biological processes, molecular function, and cellular component, as well as Reactome and KEGG pathway databases (Kanehisa 2000; Kanehisa et al. 2025). FDR was used to control for multiple testing. A threshold of 0.01 (FDR < 0.01) was used to identify significantly enriched GO terms, while 0.1 (FDR < 0.1) was used for Reactome and KEGG pathways. Semantic similarity distance as implemented in the rrvo R package (https://ssayols.github.io/rrvgo) was implemented to reduce redundancy of the significant GO terms. Maps of the significantly enriched KEGG pathways were colored according to the logarithmic fold change (log_2_FC) of the proteins using pathview R package.

### 2.11 Comprehensive Analysis of *KCNN4* Expression

The UALCAN (ualcan.path.uab.edu) platform was used to analyze the expression of the *KCNN4* gene in pancreatic cancer samples based on TCGA‐PAAD RNA‐seq data.

This analysis included comparisons between normal tissues (*n* = 4), tumors without nodal involvement (N0, *n* = 49), and tumors with lymph node metastasis (N1, *n* = 124).

Statistical comparisons were performed using the Kruskal–Wallis test followed by Dunn's post hoc test for pairwise comparisons. The resulting boxplots and *p* values provide insights into the relationship between KCNN4 expression and nodal metastatic status in pancreatic cancer (Chandrashekar et al. 2017, 2022).

Correlation analysis of gene expression was performed using GEPIA (Gene Expression Profiling Interactive Analysis), an online platform that integrates RNA sequencing data from the Cancer Genome Atlas (TCGA) and Genotype‐Tissue Expression (GTEx) projects (http://gepia.cancer-pku.cn/detail.php?clicktag=correlation###) (Tang et al. 2017). The primary data set utilised in this study was the TCGA PAAD tumour data set.

Statistical significance was assessed by calculating Pearson's correlation coefficient (*R*) and the corresponding *p* value, with correlations reaching *p* < 0.05 considered statistically significant. The strength of the correlation was categorised as follows: strong when *R* > 0.5, moderate when 0.3 < *R* < 0.5, and weak when *R* < 0.3. Scatter plots were generated through GEPIA, which employs a log‐scale transformation to gene expression values with a view to enhancing visualisation and facilitating data interpretation.

Additionally, the TNMplot (https://tnmplot.com/analysis/) tool was used to further validate the results by comparing *KCNN4* expression levels across normal, tumor, and metastatic samples. This additional analysis was conducted to confirm the trends observed in UALCAN using an independent data set (Bartha and Győrffy 2021).

### 2.12 Survival Analysis

Overall Survival (OS) was analyzed to evaluate the predictive potential of *KCNN4* expression. The survminer (v0.4.9) and survival (v3.6‐4) R packages were used to perform the log‐rank test and generate Kaplan–Meier (KM) plots for groups stratified by *KCNN4* expression levels.

### 2.13 Immunogenomic Analysis of *KCNN4* Expression and Immune Infiltration

A curated list of immune‐related genes was obtained from TISIDB (http://cis.hku.hk/TISIDB/index.php) (Ru et al. 2019). To explore the relationship between *KCNN4* expression and immune‐related genes, as well as immune infiltration levels, Pearson correlation analysis was conducted. The results were visualized using the ggplot2 (v3.5.1) R package. Additionally, the estimate (v1.0.13) R package was employed to calculate immune, stromal, and ESTIMATE scores for each sample. Pearson correlation analysis was further used to assess the association between *KCNN4* expression and these immune‐related scores. The correlation results were presented using ggplot2 (v3.5.1) and ComplexHeatmap (v2.20.0) R packages for better graphical interpretation.

The Gene Set Cancer Analysis (GSCA, https://guolab.wchscu.cn/GSCA/#/) platform, an integrated tool for genomic, pharmacogenomic, and immunogenomic cancer analysis, was used to investigate the immunogenomic landscape associated with *KCNN4* expression. This platform provided insights into the correlation between *KCNN4* expression and immune cell infiltration, copy number variations (CNVs), and mutations, offering a comprehensive immunogenomic perspective (Liu et al. 2023).

A list of autoimmune disease‐associated risk genes was collected from EMBL‐EBI databases (https://www.ebi.ac.uk/gwas/efotraits/EFO_0005140).

### 2.14 Comprehensive Analysis of Membrane Proteins Using EBI‐GO_Membrane and Membranome Databases

To explore the role of membrane proteins in various cellular processes, data were obtained from the EBI‐GO_Membrane and Membranome databases (https://membranome.org/proteins and https://www.ebi.ac.uk/QuickGO/term/GO:0016020). These databases provide a comprehensive resource for studying the structural, functional, and localization aspects of membrane‐associated proteins.

### 2.15 MitoCarta Comparison Analysis

To investigate the mitochondrial localization of K_Ca_3.1‐interacting proteins, a comparative analysis was performed using the proteins identified through TurboID‐based BioID proximity labeling in the present study and those cataloged in the Mouse MitoCarta3.0 (https://www.broadinstitute.org/mitocarta) (Rath et al. 2021).

### 2.16 Artificial Intelligence Tool

A generative artificial intelligence tool was used for language editing and stylistic improvements of the manuscript, without influencing the scientific content or methodology.

## 3 Results

### 3.1 Identification of K_Ca_3.1 Interactome Using Proximity Labeling: A Focus on Channel‐Related Signaling in PDAC Cells

**Figure 1:**
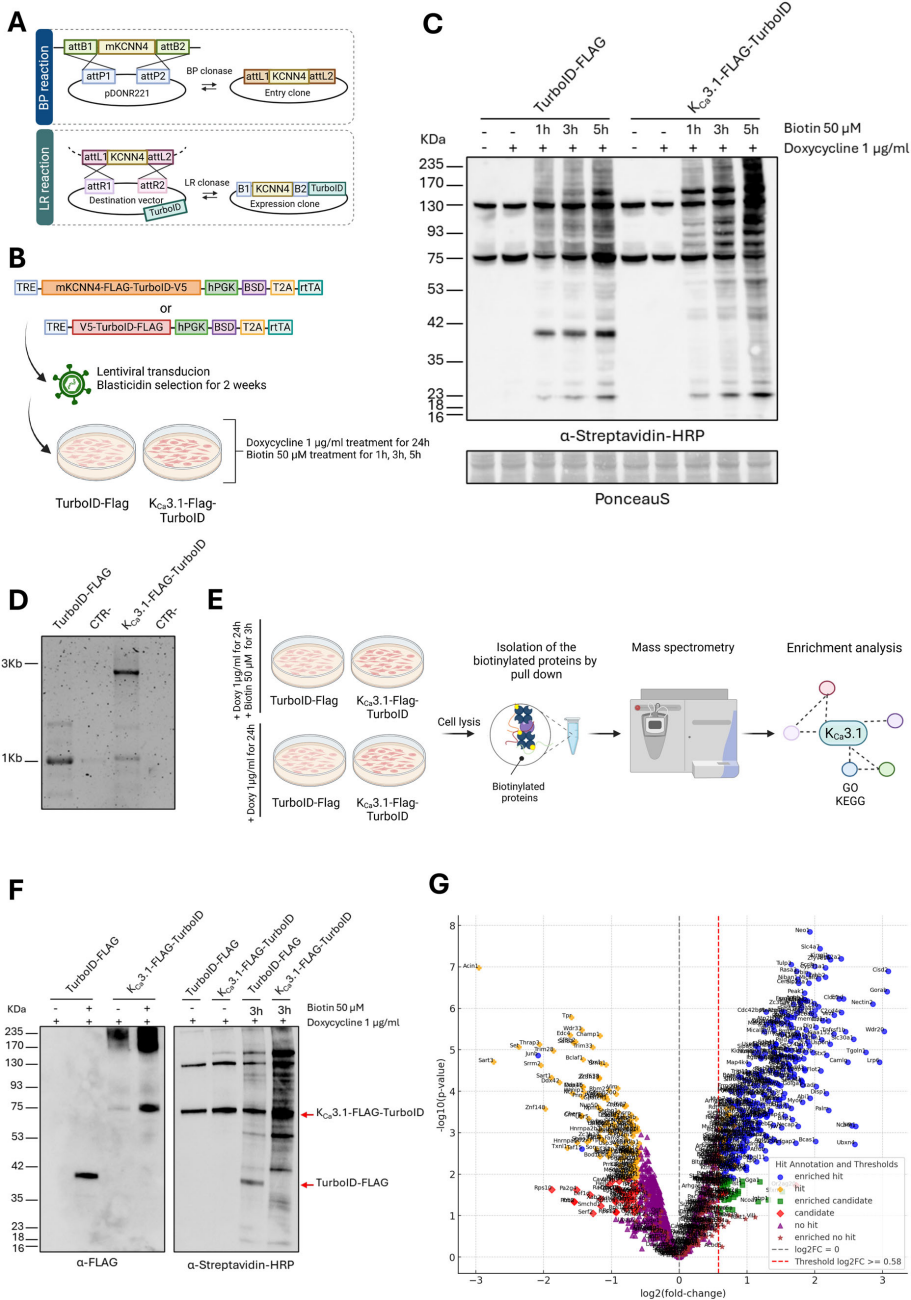
Biotinylation‐based proximity labeling and protein interaction analysis. (A) Cloning strategy for TurboID‐based proximity labeling. The BP reaction transfers the m*kcnn4* gene from the pDONR221 vector to the entry clone using BP clonase. The LR reaction integrates m*Kcnn4* into the destination vector, creating the final expression clone containing m*Kcnn4*‐TurboID for lentiviral expression. Lentiviral transduction and experimental setup. (B) Cells were transduced with either m*Kcnn4*‐FLAG‐TurboID‐V5 or V5‐ TurboID‐FLAG, followed by blasticidin selection. Cells were treated with doxycycline (1 μg/mL for 24 h) to induce expression, followed by biotin labeling (50 μM for 1 h, 3 h, or 5 h) to capture proximal proteins for further analysis. (C) Western blot analysis showing biotinylated proteins detected with α‐Streptavidin‐HRP in TurboID‐Flag and mK_Ca_3.1‐Flag‐TurboID expressing cells at different time points (1 h, 3 h, 5 h). Ponceau staining was used as a loading control. (D) PCR validation of transgene integration in the different cell lines. (E) Experimental workflow for biotinylation‐based proximity labeling and protein identification. Cells expressing TurboID vectors were treated with doxycycline (1 μg/mL for 24 h) to induce expression, followed by biotin labeling (50 μM for 3 h). After cell lysis, biotinylated proteins were isolated via streptavidin pull‐down, analyzed by MS, and subjected to GO/KEGG enrichment analysis to identify functional interactions. (F) Validation of protein expression and biotinylation efficiency. Western blot analysis using α‐FLAG confirms expression of TurboID‐tagged constructs. α‐Streptavidin‐HRP detection shows biotinylated proteins upon biotin treatment, with increased biotinylation observed in K_Ca_3.1‐Flag‐TurboID samples compared to controls. (G) Volcano Plot. The plot categorizes proteins into different groups based on their statistical significance and fold‐change: Enriched hits (blue circles): proteins with high statistical significance (*p* < 0.05) and positive fold‐change (log2FC > 0.58). Hits (orange circles): proteins that meet statistical thresholds but have lower fold‐changes. Enriched candidates (green squares): proteins with moderate fold‐change and significance (*p* < 0.05; 0.3 < log2FC ≤ 0.58). Candidates (red diamonds): potentially relevant proteins that do not meet strict thresholds. No hits (purple triangles): proteins with no significant differential biotinylation. Proteins with no significant differential expression that are still plotted (brown stars). Thresholds: The black dashed line represents log2FC = 0 (no change).

To characterize the K_Ca_3.1 interactome, inducible Tet‐On TurboBirA* control cell lines were established alongside Tet‐On K_Ca_3.1‐TurboBirA* expressing cell lines (Figure 1A,B), to avoid possible artefacts in the interactome determination due to a constantly present high expression of the channel. In the present study, we used as the parental model KPC cells that originate from the KPC (Kras^^G12D/+^; Trp53^^R172H/+^; Pdx1^−Cre^) genetically engineered mouse model (GEMM) of PDAC, a system that closely recapitulates the aggressive and metastatic behavior of human pancreatic cancer. KPC cells serve as a robust tool for investigating tumor biology, mechanisms of drug resistance, and therapeutic responses (Lee et al. 2016). TurboBirA* was fused to the C‐terminal part of the protein to avoid fusion of TurboBirA* to the N‐terminus, which is crucial for channel assembly and trafficking (Jones et al. 2004) (Figure 1B). Following administration of 1 µg/mL doxycycline, the induction of both the Flag‐TurboBirA* control and the K_Ca_3.1‐Flag‐TurboBirA* fusion protein was observed, thus demonstrating the efficacy of the inducible expression system. The successful expression of these proteins and the biotinylation were further confirmed through Western blot analysis and PCR (Figure 1C,D). Following induction, the cells were lysed, and biotinylated proteins were selectively enriched using streptavidin beads. The purified biotinylated proteins from both control and K_Ca_3.1‐Flag‐TurboBiraA* expressing cells were analyzed using quantitative mass spectrometry (MS) with tandem mass tag (TMT) labelling to enable precise relative quantification (Figure 1E,F). A comprehensive overview of normalized TMT reporter ion intensities across all analyzed samples from the three independent biological replicates is presented in Supporting Information S1: Figure S1. The volcano plot provides a graphical representation of biotinylated proteins, displaying their log₂(fold‐change) on the x‐axis and −log₁₀(*p* value) on the y‐axis (Figure 1G).

### 3.2 Proteomic Analysis: Analysis of the Proximal Protein Environment of K_Ca_3.1

**Figure 2:**
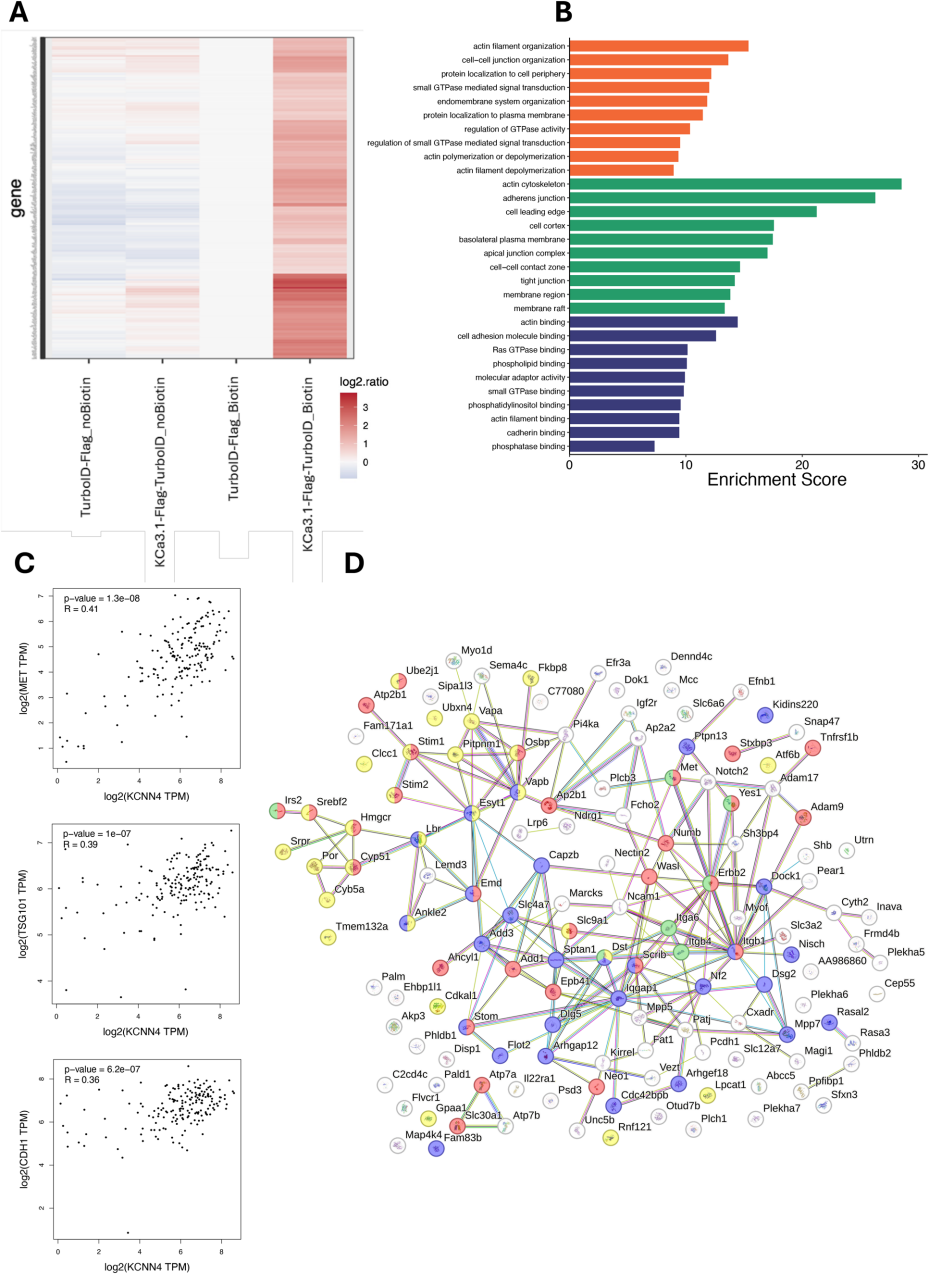
Functional enrichment and protein–protein interaction network analysis of identified K_Ca_3.1 interactors. (A) Heatmap displaying the log2 ratio of gene expression across different experimental conditions. The color scale represents expression changes, where red indicates higher expression levels (log2 ratio > 0) and blue represents lower expression levels. The biotinylated conditions show a marked increase in expression for specific genes compared to the no‐biotin conditions (refer to the attached S2 for high resolution table). (B) Gene Ontology (GO) enrichment analysis highlighting the most significant Biological Processes (BP), Cellular Components (CC), and Molecular Functions (MF) associated with the data set. The enrichment score quantifies the relative representation of each GO term. (C) Correlation between KCNN4 expression and key interactors in pancreatic cancer using correlation analysis. Scatter plots illustrate the correlation between log2(KCNN4 TPM) and the expression levels (log2(TPM)) of selected interacting proteins. The Pearson correlation coefficient (*R*) and *p* value for each correlation are displayed in the plots. A significant positive correlation was observed with MET (*R* = 0.41, *p* = 1.3e−08), TSG101 (*R* = 0.39, *p* = 1e−07), and CDH1 (*R* = 0.36, *p* = 2.6e−07). (D) PPI network of the 138 membrane proteins identified among the interactors. Functional enrichment analysis of STRING MP network. The figure highlights key biological processes and signaling pathways associated with the analyzed data set. GO:0051049—Regulation of transport (red): 29 of 1998 genes; enrichment score = 0.36; fold change = 0.43; *p* value = 0.0047. GO: 0005789 Endoplasmic reticulum membrane (yellow): 27 of 1100 genes; enrichment score = 0.59; fold change = 1.0; *p* value = 1.09e−07. ReacTome: MMU‐194315—Signaling by Rho GTPases (blue): 28 of 603 genes; enrichment score = 0.87; fold change = 1.86; *p* value = 1.89e−13. WP488–Alpha 6 beta 4 integrin signaling pathway (green): 7 of 66 genes; enrichment score = 1.23; fold change = 1.04; *p* value = 7.22e−05. Each pathway is color‐coded according to its classification, indicating significant involvement in cellular signaling and transport regulation.

To ensure a thorough characterization of the proximal protein environment surrounding the K_Ca_3.1 channel, a rigorous filtering criterion was implemented in the data set. Only proteins identified with at least two unique peptide matches were retained for further analysis, ensuring a high‐confidence data set. The assessment of differential protein abundance was facilitated by the implementation of a moderated t‐test, utilizing the limma R package. This approach employed a false discovery rate (FDR) threshold of < 0.05 and a minimum fold‐change (FC) of 1.5‐fold to determine statistical significance. The resulting heatmap provides a visual representation of the log₂‐transformed enrichment ratios of diverse proteins across four distinct experimental conditions. These conditions are labelled as follows: TurboID‐FLAG_noBiotin, K_Ca_3.1‐FLAG‐TurboID_noBiotin, TurboID‐FLAG_Biotin, K_Ca_3.1‐FLAG‐TurboID_Biotin. The color scale represents relative protein enrichment, where red indicates increased abundance, while blue indicates depletion relative to the control condition (Figure 2A, see Supporting Information S1: Figure S2 for enlarged image and Supporting Information S2: Table 1). Conditions devoid of biotin (referred to as “noBiotin”) exhibited negligible changes in protein abundance, thereby substantiating the conclusion that the observed enrichment is exclusively contingent on biotinylation and not on nonspecific interactions. In addition, the TurboID‐FLAG_Biotin condition displays comparatively low enrichment levels, suggesting that biotinylation predominantly occurs in the presence of K_Ca_3.1‐FLAG‐TurboID_Biotin, rather than through endogenous biotinylation pathways (Figure 2A). Conversely, the significant enrichment pattern observed in K_Ca_3.1‐FLAG‐TurboID_Biotin further supports the hypothesis that these proteins are directly or indirectly associated with K_Ca_3.1. Overall, we identified 283 K_Ca_3.1‐specific interactor proteins, providing valuable insights into the K_Ca_3.1 interactome and its potential functional network (Figure 2A). A range of functional categories can be distinguished among these proteins, including those involved in cell adhesion and junctions, cytoskeletal regulation, signaling pathways, vesicular trafficking, and drug resistance (see Supporting Information S2: Table 1 for the complete list of identified proteins).

### 3.3 Defining the K_Ca_3.1 Interactome in Living Cells and Its Role in Cellular Regulation

To assess the relevance of the findings of the BioID experiments, gene set analysis was conducted. Pathway enrichment analysis was performed using Gene Ontology (GO) and Pathway Reactome (PR) to examine the BioID results that had been compiled. The GO analysis of BioID‐identified proteins revealed significant functional enrichment across three main ontologies: Molecular Function (MF), Cellular Component (CC), and Biological Process (BP). The top 10 significantly enriched terms (*p *< 0.05) in each category are displayed in Figure 2B. These provide insights into the functional roles of the identified interactors, highlighting key molecular activities, subcellular localizations, and biological pathways associated with the K_Ca_3.1 interactome. The analysis of molecular functions indicates a strong enrichment for proteins involved in protein binding and signalling regulation. Among the most prominent functions, phosphatase binding, cadherin binding, and actin filament binding suggest interactions with cytoskeletal and adhesion‐related proteins, while phosphatidylinositol binding and phospholipid binding imply roles in lipid signaling and membrane dynamics. Furthermore, the enrichment of small GTPase binding, Ras GTPase binding, and molecular adaptor activity underscores the involvement of K_Ca_3.1 interactors in intracellular signal transduction and regulatory networks (Figure 2B). The subcellular localization of enriched proteins aligns with their functional roles, as indicated by their association with membrane rafts, membrane regions, and the actin cytoskeleton, suggesting interactions with membrane‐associated and cytoskeletal structures. Furthermore, their presence in tight junctions, adherens junctions, and the apical junction complex underscores their role in cell adhesion and intercellular communication. The localization of these proteins to the cell leading edge and cell cortex serves to reinforce the hypothesis that K_Ca_3.1 and its associated proteins contribute to cell motility and structural organization (Figure 2B). The biological processes enriched in the data set provide further evidence for the role of K_Ca_3.1 in cellular regulation. This is indicated by the presence of proteins involved in actin filament organization, polymerization, and depolymerization, suggesting a direct contribution to cytoskeletal remodeling. Additionally, the enrichment of processes related to the regulation of small GTPase‐mediated signal transduction and GTPase activity emphasizes the influence of K_Ca_3.1 on intracellular signaling pathways. These data also indicate a significant role in protein localization to the plasma membrane and the cell periphery, supporting its association with membrane‐bound signaling pathways. Finally, the enrichment of terms related to cell–cell junction organization, endomembrane system organization, and cell adhesion molecule binding highlights the involvement of K_Ca_3.1 in cellular communication and structural integrity (Figure 2B). In agreement with the localization of this channel to mitochondria as well, some of the K_Ca_3.1 interactors were mitochondrial proteins, such as SUCLA2, IARS2, SFXN3, STOM, AHCYL1, and FKBP8. Among these interactors, SFXN3 and IARS2, as well as STOM showed positive and negative correlation with K_Ca_3.1 in PAAD, respectively (Supporting Information S1: Figure S3). Interestingly, the correlation analysis presented in (Figure 2C) highlights significant associations between K_Ca_3.1 (encoded by *KCNN4*) expression and some of the key proteins, in part identified as interactors of human K_Ca_3.1 in the BioGRID database (https://thebiogrid.org) (Balut et al. 2010; Guo et al. 2014; Huttlin et al. 2021), involved in tumour progression, signalling, and cellular adhesion in PAAD. Notably, TSG101, CDH1, and MET exhibit strong positive correlations with *KCNN4*, suggesting their potential coregulation or involvement in common oncogenic pathways. These findings reinforce the potential role of *KCNN4* as a modulator of tumor biology, supporting its relevance as a therapeutic target in PAAD.

To further explore the regulation of *KCNN4* expression in cancer, we analyzed its association with common driver gene mutations in PAAD and colorectal adenocarcinoma (COAD) (Supporting Information S1: Figure S4). As shown in Supporting Information S1: Figure S4, violin plots illustrate *KCNN4* expression stratified by the mutational status of key driver genes, including KRAS, CDKN2A, SMAD4, and BRCA2. In PAAD, *KCNN4* expression was significantly elevated in tumors harboring KRAS mutations (p = 3.5e−16) and CDKN2A mutations (*p* = 2.1e−05), suggesting a possible link between *KCNN4* and oncogenic signaling pathways. No significant differences in *KCNN4* expression were observed in association with SMAD4 (*p* = 0.093) or BRCA2 (*p* = 0.86) mutations. Similarly, in COAD samples, KRAS mutations were associated with significantly higher *KCNN4* expression (*p* = 1.3e−09), whereas no statistically significant changes were detected for CDKN2A (*p* = 0.1), SMAD4 (*p* = 0.06), or BRCA2 (*p* = 0.15). These findings suggest a consistent association between KRAS mutation status and increased *KCNN4* expression across distinct gastrointestinal malignancies. Statistical analysis was performed using the Wilcoxon rank‐sum test.

The 138 membrane‐associated interactors are primarily involved in transport regulation, ER membrane localization, Rho GTPase signaling, and integrin‐mediated signaling, suggesting key roles in cellular trafficking, adhesion, and cytoskeletal dynamics (Figure 2D).

The KEGG pathway analysis furthermore reveals a correlation between potassium channel activity and key cellular processes and signaling pathways, including endocytosis pathway (Figure 3A), adherens junctions pathway (Figure 3B), RAP1 signaling pathway (Figure 3C) and calcium signaling pathway (Figure 3D). These findings underscore the role of K_Ca_3.1‐linked ion homeostasis and transport in PDAC progression, invasion, and therapy resistance.

Figure 3KEGG pathway enrichment analysis of K_Ca_3.1 BioID interactors. (A) Endocytosis pathway. (B) Adherens junctions pathway. (C) RAP1 signaling pathway. (D) Calcium signaling pathway.

**Figure:**
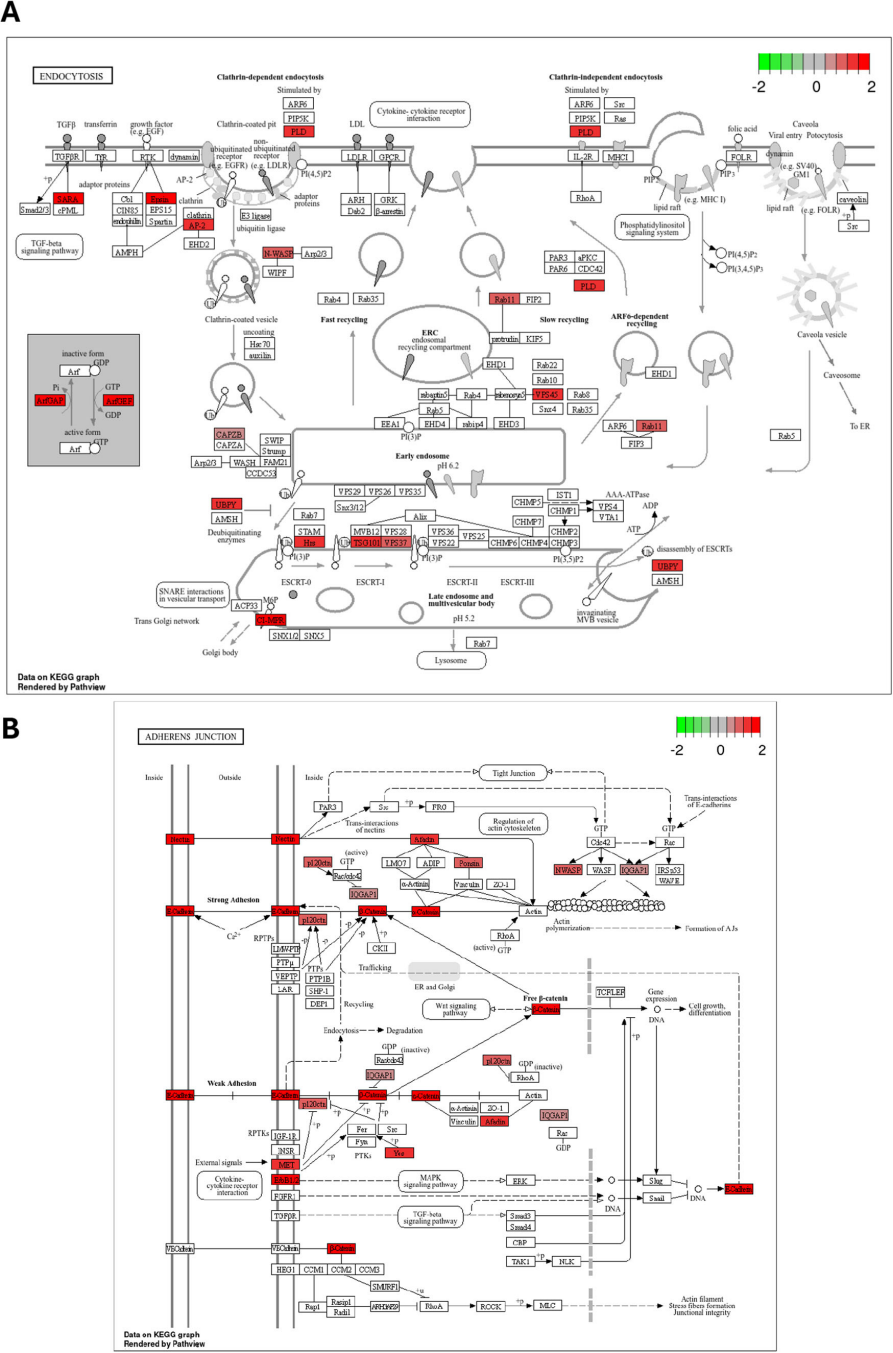

**Figure:**
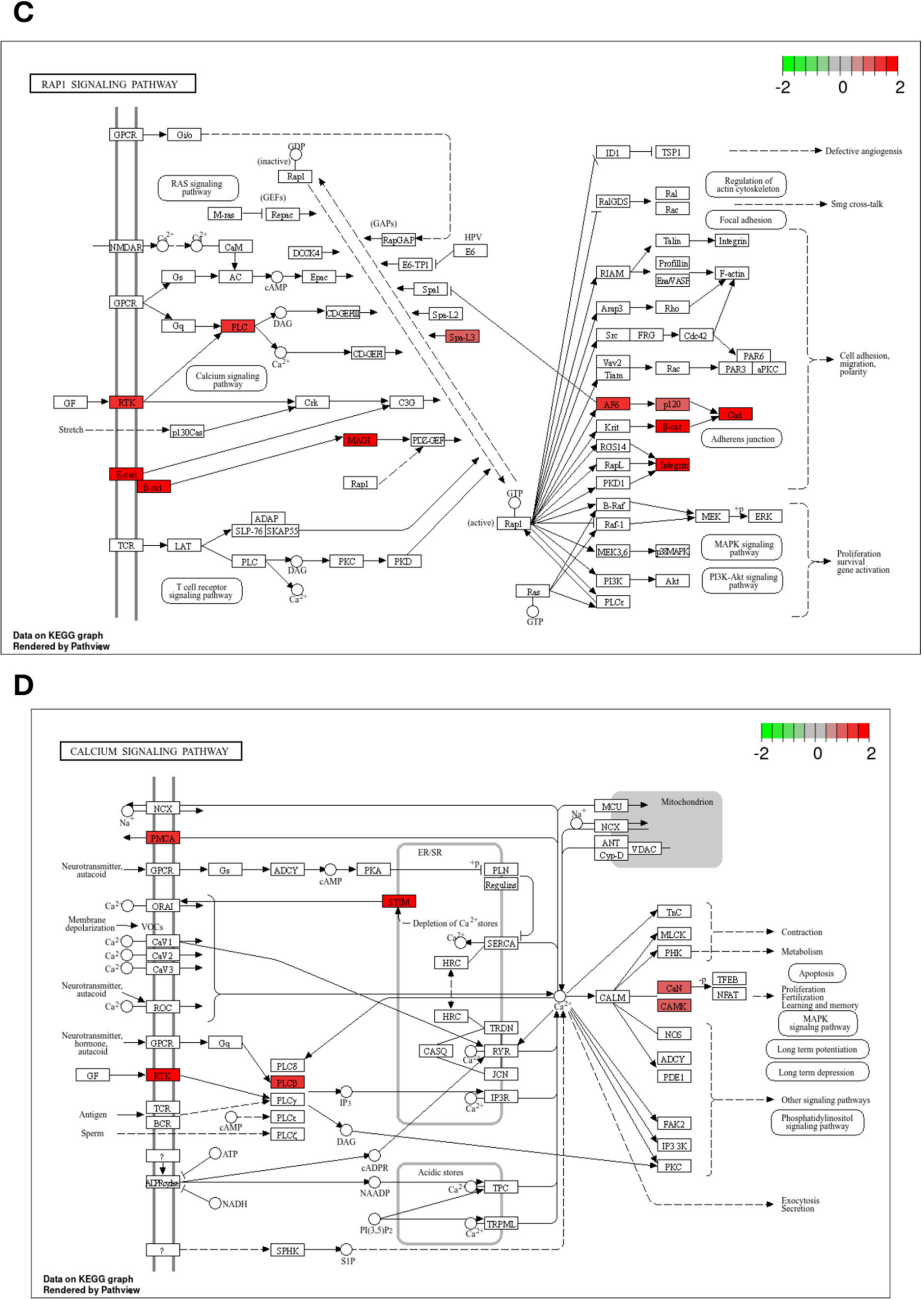

### 3.4 Biochemical Validation of Some K_Ca_3.1 Interactors Identified by BioID

**Figure 4:**
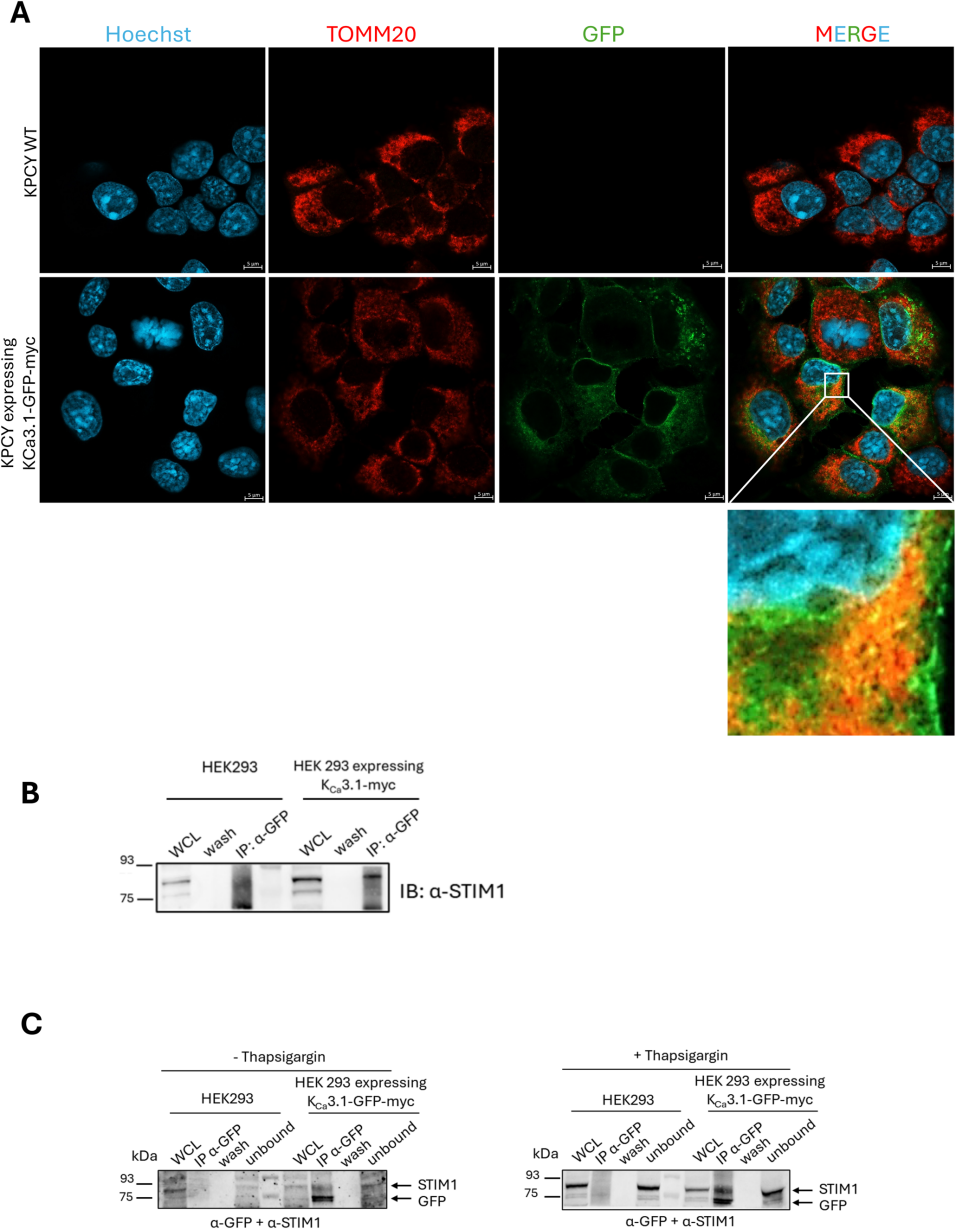
Biochemical validation of interactors of K_Ca_3.1 uncovers novel functional roles of the channel in the regulation of cellular dynamics. (A) Immunofluorescence analysis of K_Ca_3.1‐GFP‐ myc subcellular localization in KPCY cells. K_Ca_3.1‐GFP‐myc‐overexpressing cells were immunostained with an anti‐TOMM20 antibody (red) to visualize mitochondria. K_Ca_3.1 is visualized in green via the GFP tag. Nuclei were counterstained with Hoechst (blue). (B) K_Ca_3.1 immunoprecipitates with STIM1 in HEK293 cell line over‐expressing K_Ca_3.1. Immunoprecipitation was performed using an anti‐GFP antibody to pull down GFP‐tagged K_Ca_3.1. Western Blot analysis revealed the presence of STIM1, indicating a physical interaction between K_Ca_3.1 and STIM1. (C) K_Ca_3.1 co‐immunoprecipitates with STIM1 under both basal and SOCE‐activated conditions, indicating that their interaction is stable independently of calcium store depletion.

Following the bioinformatic analysis of the BioID results, we aimed to validate some of the interesting, novel interactions with K_Ca_3.1 by biochemical methods. First, we generated HEK293 and KPCY cell lines stably expressing the channel and confirmed the localization of K_Ca_3.1 to both the plasma membrane and mitochondria. The channel localized to both cellular compartments, as expected—in particular, a part of the channel protein colocalized with both mitoTracker Red and TOMM20, indicating mitochondrial localization in both cell lines (Figure 4A and Supporting Information S1: Figure S5A–C). Next, to provide further evidence of specific interaction with one of the unexpected hits of our proximity labeling analysis, we performed co‐immunoprecipitation using the anti‐tag (GFP) antibody in both non‐transfected and K_Ca_3.1‐GFP‐myc‐transfected HEK293 cells, which do not express endogenously this channel. STIM1, located in the endoplasmic reticulum was one of the unexpected hits in our BioID analysis, although the IP samples were therefore evaluated using anti‐STIM1 antibody, revealing a strong interaction between the two proteins only in the channel‐expressing cells (Figure 4B). This interaction was also observable upon incubation of the cells with thapsigargin, an inhibitor of sarco/endoplasmic reticulum Ca^2+^‐ATPase (SERCA), known to trigger store‐operated calcium entry (SOCE) in a STIM1‐ and Ora1‐dependent manner in PDAC cells (Kondratska et al. 2014) (Figure 4C). However, under our experimental conditions, Co‐IP did not reveal interaction of K_Ca_3.1 with Orai1 (Supporting Information S1: Figure S6A).

**Figure 5:**
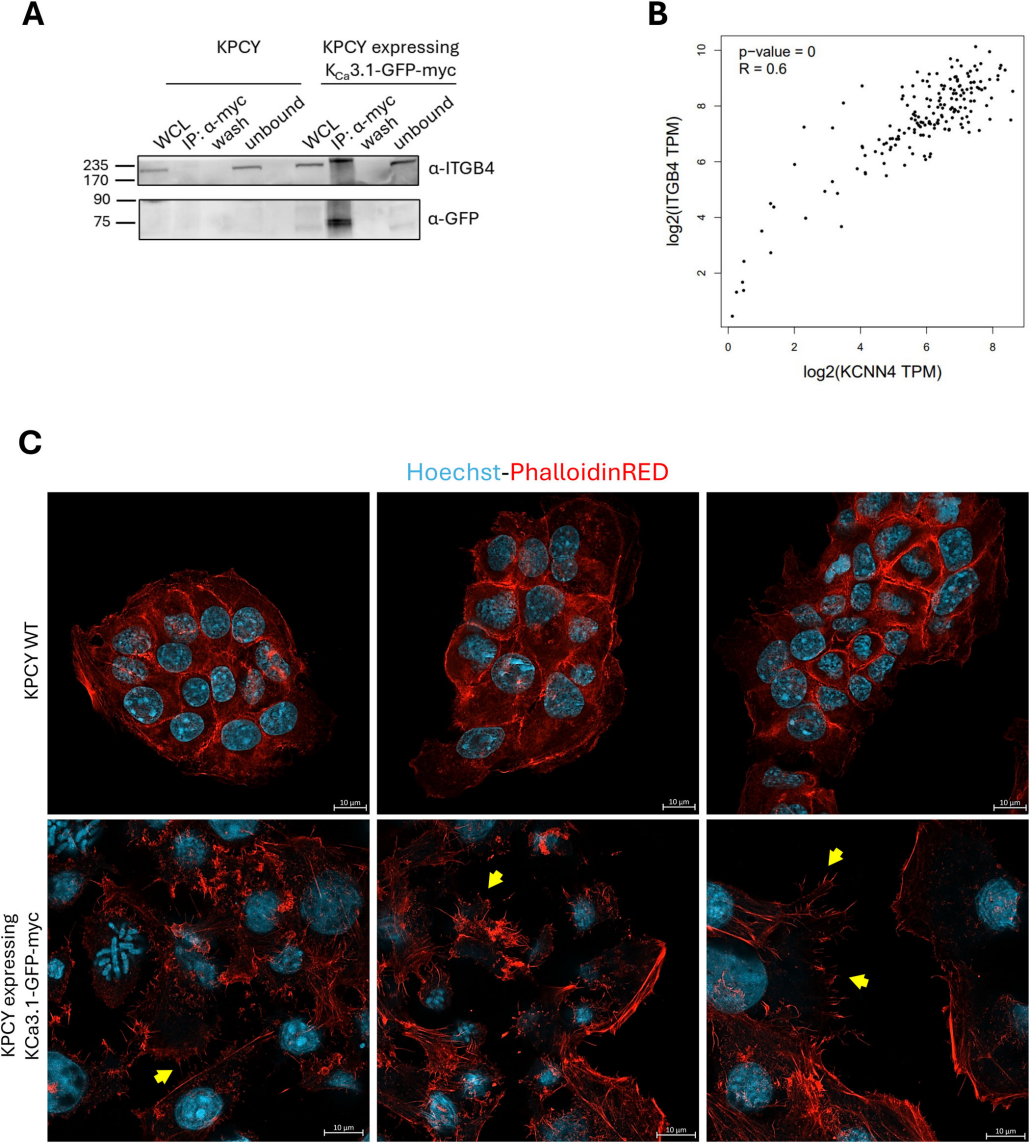
K_Ca_3.1 physically interacts with ITGB4 and promotes cytoskeletal remodeling: (A) K_Ca_3.1 immunoprecipitates with ITGB4 in K_Ca_3.1 over‐expressing cell line. Immunoprecipitation was performed using an anti‐myc antibody to pull down myc‐tagged K_Ca_3.1 in KPCY cells. Western Blot analysis revealed the presence of ITGB4, indicating a physical interaction between K_Ca_3.1 and ITGB4. (B) Gene expression correlation analysis between KCNN4 and ITGB4. Scatter plot illustrating a significant positive correlation between KCNN4 and ITGB4 expression levels (Pearson's *R* = 0.6, *p* < 0.0001), indicating a potential coregulation or functional association between the two genes. (C) Confocal images of phalloidin staining in KPCY cells over‐expressing K_Ca_3.1‐GFP‐myc. Overexpression of K_Ca_3.1 in KPCY cells leads to actin cytoskeletal remodeling, characterized by multiple filopodial extensions and well‐defined stress fibers that, in contrast, are largely lacking in the KPCY WT cell line.

Another so far unknown interactor revealed by our BioID analysis was integrin‐β4, a protein that could be immunoprecipitated with antibodies against K_Ca_3.1‐GFP in KPC cells (Figure 5A) and a significant correlation between the expression of the two genes can be detected in PDAC (Figure 5B). Given that high integrin β4 expression was shown to promote epithelial‐mesenchymal transition, stabilize filopodia and represents a prognostic marker in pancreatic ductal adenocarcinoma (Masugi et al. 2015), we tested whether filipodia formation was increased upon overexpression of the interacting K_Ca_3.1 channel, showing that overexpression of the channel in KPCY cells led to actin cytoskeletal remodeling, characterized by multiple filopodial extensions and well‐defined stress fibers (Figure 5C).

Finally, given that at least a part of the channel localizes to mitochondria, and a few mitochondrial partners have been identified by BioID, we performed correlation analyses (Supporting Information S1: Figure S6B) and Co‐IP for SUCLA2, IARS2, SFXN3, STOM and FKBP8 but could not reveal strong interactions (Supporting Information S1: Figure S6C,D).

### 3.5 Expression of *KCNN4* in Pancreatic Cancer: An Integrated Analysis

**Figure 6:**
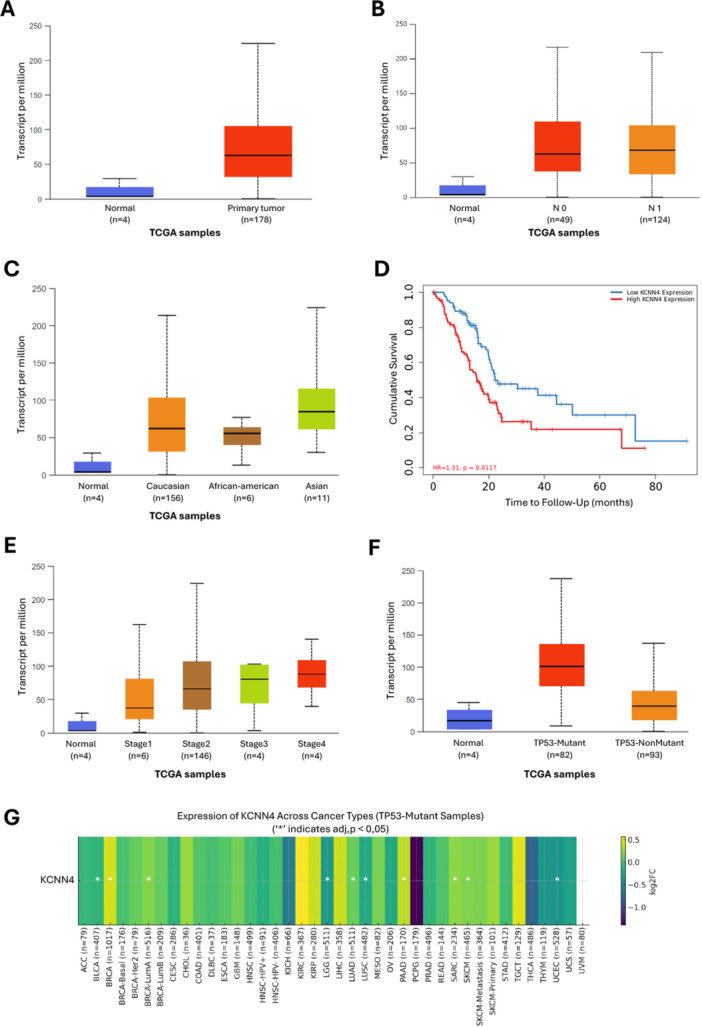
Expression of the *KCNN4* gene in tumor and normal samples from the TCGA‐PAAD data set: association with disease progression and survival outcomes. (A) KCNN4 expression in TCGA‐PAAD RNA‐seq data: comparison between normal tissues (*n* = 4, blue) and primary tumor samples (*n* = 178, red). (B) KCNN4 expression in TCGA‐PAAD samples stratified by nodal metastasis status: normal (*n* = 4), N0 (*n* = 49), and N1 (*n* = 124). (C) KCNN4 expression stratified by ethnicity: Caucasian (*n* = 156), African American (*n* = 6), and Asian (*n *= 11), compared to normal samples (*n* = 4). (D) Kaplan–Meier survival analysis of TCGA‐PAAD patients stratified by KCNN4 expression (high vs. low); high expression is associated with significantly worse prognosis (*p* = 0.0117, HR = 1.31). (E) KCNN4 expression across tumor stages I–IV in TCGA‐PAAD, compared to normal tissues. (F) KCNN4 expression in TCGA‐PAAD samples with TP53 mutation (*n* = 82), non‐mutated TP53 (*n* = 93), and normal tissues (*n* = 4). (G) TP53 mutations are associated with elevated KCNN4 expression across multiple cancer types. Heatmap shows the log2 fold change in KCNN4 expression in TP53‐mutant cancers from TCGA; asterisks (*) indicate statistically significant differences (adjusted *p* < 0.05). Statistical comparisons in panels (A, B, C, E, and F) were performed using the Kruskal–Wallis test, followed by Dunn's post hoc test for multiple pairwise comparisons. Sample numbers are indicated below each group. Abbreviation: TPM, Transcripts Per Million.

Given the fact that we performed our interactome analysis in pancreatic cancer cells and the results pointed to the involvement of K_Ca_3.1 in several cancer‐related pathways, we aimed to integrate the results of our BioID analysis with information available in public databases. Although an initial Pan‐cancer analysis of *KCNN4* expression in various tumors has been reported 3 years ago (Chen et al. 2022), in the rapidly evolving domain of cancer research, the periodic re‐examination of bioinformatics data following a period of several years is imperative to ensure the accuracy, relevance, and completeness of findings. Indeed, our analysis revealed a clear distinction between normal and tumor *KCNN4* expression in PAAD, a result that was less evident in the study of Chen and colleagues (Chen et al. 2022) due to the absence of samples from normal healthy tissues. Analysis of TCGA‐PAAD RNA‐seq data reveals that KCNN4 expression is significantly elevated in pancreatic tumor samples compared to normal tissues (*p* < 3.36e−26) (Figure 6A), and also shows increased expression in tumors with nodal metastasis compared to non‐metastatic tumors (Figure 6B). This increase is clearly observed in both bulk RNA‐seq data from TCGA and Gene Chip analyses, thus reinforcing the hypothesis that K_Ca_3.1 encoded by *KCNN4* plays a relevant role in pancreatic cancer progression in humans. *KCNN4* expression analysis across ethnic groups reveals a significantly higher upregulation in Asian patients' cancer tissues compared to normal ones (*p* = 0.038), while no significant differences are observed between PAAD tissues from Caucasian, African American, and normal tissue samples. This may indicate potential ethnicity‐specific variations in *KCNN4* regulation. However, the limited sample size in non‐Caucasian groups reduces the robustness of this observation, necessitating further studies to validate these findings (Figure 6C). The analysis of *KCNN4* expression at varying tumor stages reveals that the most substantial increase occurs in advanced‐stage tumors (Stage 4, *p*= 0.031), pointing to a prominent role of the channel in the late, metastatic phases. Additionally, TCGA‐PAAD data stratified by nodal metastasis status indicates higher KCNN4 expression in tumors with lymph node involvement (N1) compared to those without (N0), suggesting a possible association between KCNN4 upregulation and local metastatic progression (Figure 6B).

The analysis of *KCNN4* expression at varying tumor stages reveals that the most substantial increase occurs in advanced‐stage tumor (Stage 4, *p* = 0.031), pointing to a prominent role of the channel in the late, metastatic phases. However, Gene Chip data indicate that metastatic samples exhibit expression levels that are either like or slightly lower than those observed in primary tumor, albeit with considerable variability. This suggests a potential role for *KCNN4* in tumor progression; however, its specific involvement in metastatic dissemination requires further investigation (Figure 6B).

The overall survival (OS) analysis in PAAD patients highlights a significant association between high *KCNN4* expression and poor prognosis. The Kaplan–Meier survival curve further demonstrates that patients exhibiting high *KCNN4* expression (red line) have reduced survival rates in comparison to those with low expression (blue line). The hazard ratio (HR) of 1.31 (*p* = 0.0117) indicates that patients with elevated *KCNN4* levels have a 31% higher risk of mortality, with a statistically significant difference (Figure 6D). Moreover, the findings suggest that *KCNN4* expression is upregulated in PAAD compared to normal tissue and remains high across different tumor stages, supporting its potential role in disease progression. (Figure 6A,B,E).

In accordance with prior evidence suggesting a functional association between *KCNN4* and KRAS signaling in colorectal cancer, our analysis revealed that *KCNN4* expression is significantly elevated in KRAS‐mutant PDAC samples compared to wild‐type (Wilcoxon *p* = 3.5 × 10⁻¹⁶). A similar and robust association was observed in colon adenocarcinoma (COAD) cohort (*p *= 1.9 × 10⁻⁹), reinforcing the hypothesis of a conserved regulatory relationship between KRAS activation and *KCNN4* expression across epithelial malignancies. Beyond KRAS, we observed that *KCNN4* expression was also significantly higher in PDAC samples with *CDKN2A* mutations (*p* = 2.1 × 10⁻⁵). This finding may reflect a broader involvement of *KCNN4* in pathways governing cell cycle regulation and tumor progression. Interestingly, this association was not replicated in the COAD cohort, likely due to the low prevalence of CDKN2A mutations in this population, which limits statistical power and interpretability.

We did not observe statistically significant differences in *KCNN4* expression in relation to *SMAD4* or *BRCA2* mutation status in either PDAC or COAD. This lack of association may reflect the lower frequency of these mutations in the data sets analyzed, or it may indicate that *KCNN4* expression is not directly modulated by pathways governed by these tumor suppressors under basal conditions.

Finally, a correlation analysis between *KCNN4* and *TP53* mutations indicates that *TP53*‐mutant tumor exhibits significantly higher *KCNN4* expression levels compared to *TP53* wild‐type tumor (*p* < 10–11). This finding suggests a potential link between *TP53* loss‐of‐function and *KCNN4* expression increase and further suggests that *KCNN4* may contribute to the increased aggressiveness observed in *TP53*‐mutant tumors (Figure 6F). We stratified *KCNN4* expression across various cancer types specifically in *TP53*‐mutant tumors. The resulting heatmap illustrates the log2 fold change in *KCNN4* expression in this subgroup, with statistically significant differences (adjusted *p* < 0.05) marked by asterisks (Figure 6G).

**Figure 7:**
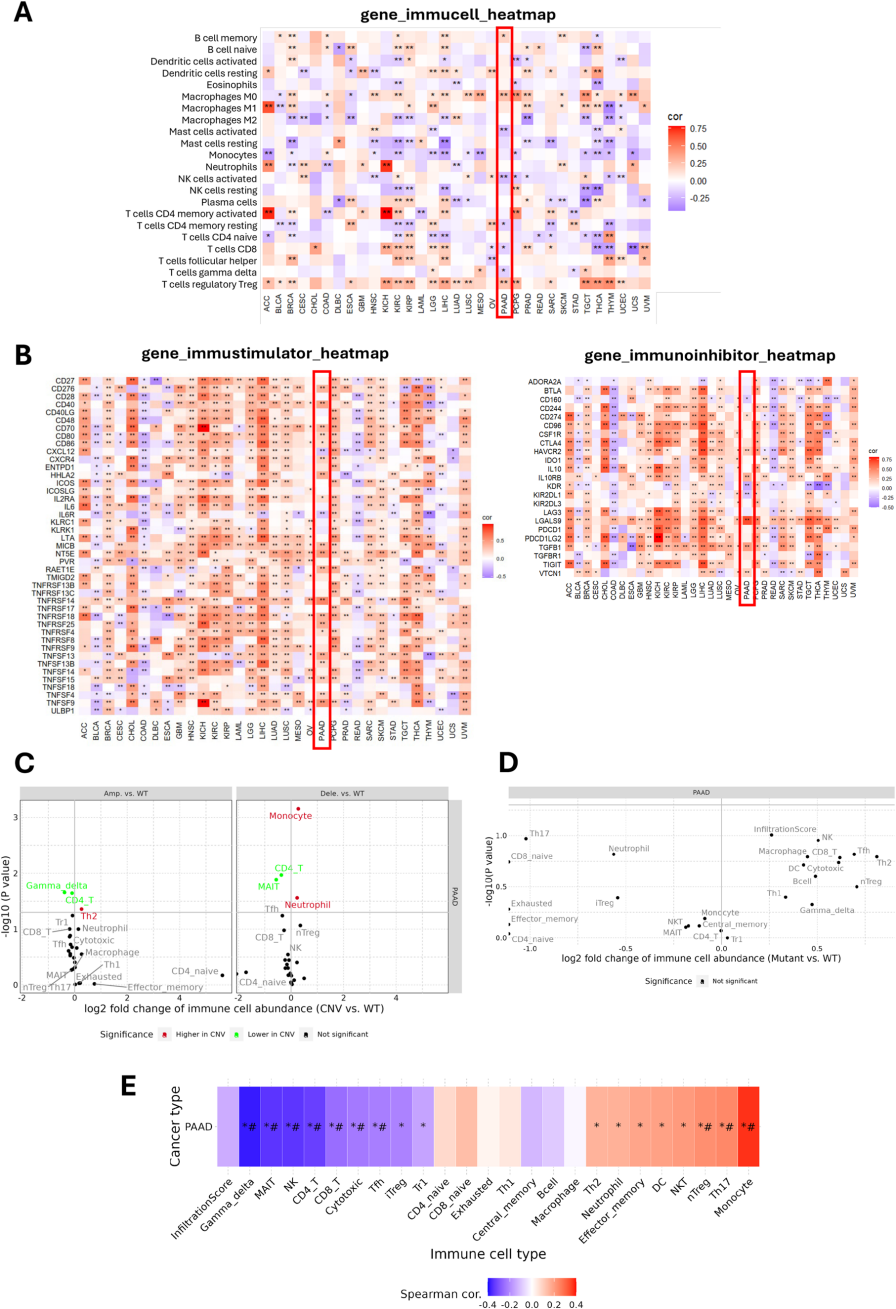
*KCNN4* Expression Correlates with Immune Cell Infiltration and Immune Modulation in PAAD. (A) Heatmap displaying correlations between *KCNN4* expression and various immune cell populations in PAAD, indicating heterogeneous immune infiltration. Positive correlations (red) indicate an association with immunosuppressive cells (e.g., Tregs, M0 macrophages), while negative correlations (blue) highlight decreased cytotoxic and effector immune cells (e.g., CD8+ T cells, NK cells). (B) Heatmaps illustrating the correlation of *KCNN4* with immune stimulatory and immune inhibitory molecules. *KCNN4* demonstrates positive associations with immune checkpoint inhibitors (e.g., PD‐L1, TIM‐3, TGFB1). (C and D) Plots depict changes in immune cell abundance in copy number variation (CNV) groups (C) and mutant vs. wild‐type (D). (E) Correlation analysis between *KCNN4* expression and immune cell infiltration in PAAD.

K_Ca_3.1 is expressed not only in the cancer cells but also in immune cells, such as T cells and macrophages (Feske et al. 2015), where the channel contributes to their activation (Cahalan and Chandy 2009). The analysis of the immune cell heatmap reveals that *KCNN4* is associated with an immunosuppressive tumor microenvironment in PAAD, as indicated by its correlation with various immune cell populations. In the gene immucell heatmap, PAAD shows variable correlations with different immune cell populations, suggesting heterogeneous immune infiltration in the pancreatic tumor (Figure 7A). *KCNN4* shows a positive correlation with memory B cells, M0 macrophages, and regulatory T cells (Tregs). The association with memory B cells suggests that *KCNN4* may contribute to the persistence of immune responses, though the role of B cells in tumors can be either supportive of immunity or involved in immune evasion. The positive correlation with M0 macrophages implies that *KCNN4* might be involved in the recruitment or maintenance of undifferentiated macrophages, which can later polarize into either pro‐inflammatory (M1) or immunosuppressive (M2) phenotypes. Furthermore, the strong correlation with Tregs indicates that *KCNN4* is linked to an immunosuppressive environment, as these cells actively inhibit antitumor immune responses by suppressing cytotoxic T cells and natural killer (NK) cell activity. In contrast, *KCNN4* demonstrates a negative correlation with mast cells, NK cells in both their activated and resting states, CD4^+^ helper T cells, cytotoxic CD8^+^ T cells, and gamma delta T cells. These cells all play pivotal roles in antitumor immunity. The negative correlation with mast cells suggests that *KCNN4* may contribute to a reduction in inflammation and antigen presentation within the tumor microenvironment. The reduced presence of NK cells implies that *KCNN4* may suppress innate immune surveillance mechanisms, thereby facilitating tumor escape from immune destruction. Furthermore, the inverse relationship with CD4^+^ and CD8^+^ T cells, along with gamma delta T cells, indicates that *KCNN4* may be involved in impairing both adaptive and innate immune responses, weakening the body's ability to mount an effective antitumor response.

The analysis of *KCNN4* correlations in the immunostimulator and immunoinhibitor heatmaps highlights its dual role in immune regulation, showing both positive and negative correlations with key immune‐related genes (Figure 7B). *KCNN4* has been shown to have positive correlations with several costimulatory molecules (CD276, CD40, CD70, CD80, CD86), immune signaling receptors (IL2RA, MICB, NT5E), and multiple TNFRSF family members, suggesting its involvement in immune modulation, inflammation, and tumor‐immune interactions. Its association with CXCR4, HHLA2, and RAET1E points to a potential role in immune cell recruitment and NK/T‐cell activation, which could contribute to an altered immune landscape in the tumor microenvironment. Furthermore, *KCNN4* demonstrates a positive correlation with immunosuppressive genes, including CD274 (PD‐L1), HAVCR2 (TIM‐3), IL10RB, LGALS9, TGFB1, and VTCN1, suggesting a potential role in immune evasion and resistance to immunotherapy.

In contrast, *KCNN4* demonstrates negative correlations with two pivotal regulators of immune cell trafficking and inflammatory signalling, namely CXCL12 and IL6R. The inverse relationship with CXCL12 suggests that *KCNN4* may reduce immune cell infiltration, particularly limiting T‐cell and dendritic cell recruitment, while its negative correlation with IL6R indicates a potential suppression of IL‐6‐driven immune responses, altering inflammatory signaling within the tumor microenvironment. In addition, *KCNN4* demonstrates a negative correlation with CD160, KDR, and KIR2DL1, which are associated with NK and T‐cell activation, as well as tumour angiogenesis. This suggests that *KCNN4* may inhibit innate and adaptive immune responses while also influencing vascular modulation. Figure 7C examines the discrepancy in immune infiltration between gene set single‐nucleotide polymorphism (SNV) groups in pancreatic cancer (PAAD), thereby demonstrating that mutations influence immune cell composition. The results indicate that CD8^+^ naive T cells and Th17 cells are enriched in WT groups, while macrophages, Tregs, NK cells, and cytotoxic T cells tend to be more abundant in mutant groups. This finding aligns with the above‐mentioned analysis, showing a positive correlation between *KCNN4* and Tregs and macrophages, and a negative correlation with CD8^+^ and NK cells. These results suggest that *KCNN4* may play a role in promoting an immune‐evasive tumor microenvironment driven by genetic mutations. The depletion of CD8^+^ T cells in mutant groups, in combination with the increase in macrophages and Tregs, supports the hypothesis that certain mutations reinforce immunosuppressive mechanisms like those influenced by *KCNN4* expression. Figure 7D focuses on immune infiltration changes in CNV groups (Amplification vs. Deletion vs. WT) in PAAD, revealing distinct immune alterations. The findings indicate that gamma delta T cells and CD4^+^ T cells are more abundant in WT compared to amplified CNVs, while Th2 cells are enriched in amplification groups, suggesting that gene amplification contributes to a shift towards an immunosuppressive phenotype. The deletion group exhibited an increase in monocytes and neutrophils, while CD4^+^ T cells and MAIT cells were more abundant in WT, indicating that gene deletions may impact innate immune responses differently. These findings align with the previous analysis of *KCNN4* negative correlation with CD4^+^ T cells and gamma delta T cells, reinforcing its role in immune suppression. Furthermore, the enrichment of Th2 cells in amplified CNV groups corroborates the observation that *KCNN4* promotes immune regulation rather than an effective antitumor response, thereby supporting its involvement in shaping an immune‐resistant tumor microenvironment. Figure 7E explores the association between GSVA scores and cancer‐related pathway activity in PAAD, showing correlations between immune cell types and oncogenic pathway activation. The results indicate that gamma delta T cells, NK cells, CD4^+^ T cells, CD8^+^ T cells, and cytotoxic T cells are negatively correlated with pathway activity, while Tregs, monocytes, and neutrophils exhibit positive correlations. This finding aligns with previous research, which reported a negative correlation between *KCNN4* and cytotoxic and innate immune cells, while observing a positive correlation with immunosuppressive populations such as Tregs and monocytes. The observed association between immune‐suppressive cell types (Tregs, neutrophils, and monocytes) and active oncogenic pathways suggests that *KCNN4* may play a role in promoting tumor progression by facilitating immune evasion and diminishing antitumor immune responses.

## 4 Discussion

### 4.1 K_Ca_3.1 and Its Proxisome

In the present study, we aimed to gain useful information about the interacting partners of K_Ca_3.1 encoded by *KCNN4*, specifically in pancreatic cancer. The number of interactors is in line with other studies providing information about K^+^ channels' proxisome (Park et al. 2020; Bowen et al. 2024; Prosdocimi et al. 2024). Six of the identified proteins here have already been reported as interactors of the human K_Ca_3.1 in the BioGRID database (https://thebiogrid.org), suggesting reliability and relevance of the detected interactions. These include CDH1, DISP1, SCFD1, USE1, TSG101 and USP8 (Balut et al. 2010; Guo et al. 2014; Huttlin et al. 2021). On the other hand, some proteins experimentally shown to interact with K_Ca_3.1 in cell types different from PDAC cells were not present in our list. These include cystic fibrosis transmembrane regulator CFTR (Klein et al. 2016), PMCA4b (plasma membrane calcium‐transporting ATPase 4b) (Allegrini et al. 2025), myotubularin‐related protein 6 (MTMR6) that function as a lipid (PI(3)P) phosphatase (Srivastava et al. 2006), and gamma‐aminobutyric acid type A receptor pi subunit (GABRP) (Jiang et al. 2019). AMP‐activated protein kinase (AMPK) has been shown to directly interact with the distal C‐terminus of K_Ca_3.1 and inhibit channel function (Klein et al. 2009). Furthermore, calmodulin C‐lobe constitutively binds to an intracellular domain of the channel to confer calcium sensitivity (Morales et al. 2013). These observations altogether point to some generalizable and to some cell‐specific features of K_Ca_3.1 interactome. Here below we discuss some of the interacting proteins and K_Ca_3.1‐linked pathways focusing on cancer/pancreatic cancer.

### 4.2 Mitochondrial Interactors of K_Ca_3.1

As mentioned above, K_Ca_3.1 is located in both the plasma membrane, and mitochondria and we identified several known interactors as well as novel proteins at both locations. Interestingly, most identified interactors were cytosolic proteins, and only few proteins out of the 283 interactors are annotated as mitochondrial proteins according to Mitocarta3.4 (Rath et al. 2021). K_Ca_3.1 has been found in mitochondria of different PDAC lines (Kovalenko et al. 2016; Bachmann et al. 2022), and its pharmacological modulation was shown to alter mitochondrial physiology (Quast et al. 2012; Kovalenko et al. 2016; Bachmann et al. 2022), although the targeting signal accounting for mitochondrial localization of this protein has not been identified. Thus, one possibility that could explain our finding is an interference of the BirA* fusion with an optimal mitochondrial localization of K_Ca_3.1. Given that the size of GFP is like that of BirA* and the K_Ca_3.1‐GFP at least in part localizes to mitochondria, this possibility is unlikely. Alternatively, K_Ca_3.1 would indeed interact only with a few mitochondrial proteins, namely FKBP8, ACHYL1, STOM, SFXN3, SUCLA2, and IARS2. These proteins have different roles, in autophagosome formation (FKB8), ion channel regulation (STOM), metabolic pathways (SUCLA2), and transport of serine into mitochondria (SFXN3). IARS2 is a mitochondrial Isoleucyl‐tRNA synthetase, while AHCYL1 is an adenosylhomocysteine hydrolase‐like protein 1, whose expression was proposed to be considered as a novel biomarker for predicting prognosis and immunotherapy response in colorectal cancer (Li et al. 2022). For this reason, we aimed to confirm interactions of the channel with these proteins, but we could not detect immunoprecipitates under our experimental conditions, suggesting either a weak or transient interaction or the necessity of using specific detergents (e.g., digitonin, i.e., often used to reveal mitochondrial protein complexes (Mokranjac 2003). Further work is required to address this point, along with a bioenergetic assessment of the cell lines overexpressing the channel.

### 4.3 Cancer‐Relevant K_Ca_3.1 Interactors

A particularly noteworthy discovery from our BioID analysis is the identification of MET, a critical tyrosine kinase receptor, as an (indirect or direct) interactor of K_Ca_3.1. The correlation analysis revealed significant associations between MET (*R* = 0.41, *p* = 1.3 × 10⁻⁸) and *KCNN4* (encoding K_Ca_3.1), reinforcing the molecular/functional link between these two proteins (Mo et al. 2022). Mo et al. demonstrated that K_Ca_3.1 knockdown, achieved through shRNA‐mediated silencing or pharmacological inhibition with TRAM‐34, significantly reduced intracellular Ca^2+^ levels in pancreatic cancer cells (Mo et al. 2022). Furthermore, treatment with BAPTA, a calcium chelating agent, resulted in a dose‐dependent suppression of MET expression and AKT phosphorylation, emphasizing the critical role of calcium signaling in this regulatory pathway. Conversely, enhancing calcium influx using 1‐EBIO, a K_Ca_3.1 activator, led to a corresponding increase in MET expression and AKT phosphorylation, further supporting the notion that calcium signaling is a key driver of K_Ca_3.1‐mediated MET regulation. Furthermore, the inhibition of cell proliferation and migration following BAPTA treatment underscores the necessity of calcium mobilization for K_Ca_3.1‐driven MET activation. Collectively, these findings suggest that K_Ca_3.1 regulates MET expression through calcium‐dependent mechanisms, thereby activating the MET/AKT signaling pathway and promoting PDAC progression, thus positioning K_Ca_3.1 as a potential upstream regulator of MET and offering new insights into PDAC pathogenesis (Mo et al. 2022).

Among the KCa3.1 interactors identified and validated by co‐immunoprecipitation, two proteins have been identified as being of relevance: integrin β4 and STIM1. The latter is an ER‐resident Ca²⁺ sensor that has been demonstrated to mediate store‐operated calcium entry (SOCE) by means of direct activation of Orai1 channels at ER‐plasma membrane junctions upon store depletion. While STIM1 has also been shown to modulate TRPC1‐containing heteromeric channels, it does not directly interact with TRPC6, which is not a canonical SOCE component. In a similar manner, TRPM2 is activated by oxidative stress and ADP‐ribose, and TRPM7, although capable of influencing intracellular Ca²⁺ homeostasis, exerts only an indirect modulatory effect on SOCE. While these channels may contribute to calcium signalling in cancer, their involvement is distinct from the classical STIM1‐Orai1‐mediated SOCE pathway.

Following ER Ca^2+^ store depletion, Ca^2+^ dissociates from STIM1, which oligomerizes and translocates to ER‐plasma membrane junctions, where it activates the channel‐forming proteins. Ca^2+^ released from the ER and Ca^2+^ taken up via SOCE activate K_Ca_3.1 channels, which provide the driving force for Ca^2+^ influx. SOCE inhibition was shown to suppress PDAC cell proliferation (Dubois et al. 2021) and patient‐derived tumor growth (Khan et al. 2020). Our results suggest that, in accordance with previous proposals, KCa3.1, Orai proteins, and STIM1 are tightly coupled (Gao et al. 2010), but the molecular details of such interaction await clarification, especially in light of the fact that K_Ca_3.1 co‐immunoprecipitates with STIM1, but not with Orai1 under the conditions used here. Interestingly, disruption of Orai1‐SK3 interaction in the rafts was shown to limit metastatic spread of breast cancer cells (Girault et al. 2011; Guéguinou et al. 2014), and STIM1 was found to be located not only in the ER but also in the plasma membrane (Debant et al. 2019). Further work is in progress in our laboratory to explore the nature and consequences of K_Ca_3.1‐STIM1 interaction. As to integrins, ITGA6 and ITGB4 are integrins that are highly expressed in pancreatic cancer stem‐like cells, contributing to tumor aggressiveness, treatment resistance, and metastatic dissemination. In particular, ITGB4 has been identified as a prognostic marker in PDAC and shown to affect filopodia stability (Masugi et al. 2015). Our results point to an exciting possibility of the regulation of ITGB4 function by K_Ca_3.1 channels, as is the case for other ion channels (e.g., Duranti et al. 2021). The relevance of such crosstalk in PDAC remains to be determined.

Among the previously known and here confirmed interactors (CDH1, DISP1, SCFD1, USE1, TSG101, and USP8), CDH1 (E‐cadherin), a critical adhesion molecule, is frequently lost or downregulated in pancreatic cancer, promoting epithelial‐to‐mesenchymal transition (EMT) and enhancing tumor invasiveness (Saito et al. 2017; Micalizzi et al. 2022). USP8 is a deubiquitinating enzyme that regulates cell signaling pathways by modulating the turnover of key receptors, including the transforming growth factor‐beta receptor II (TβRII). Recent studies have demonstrated that USP8‐mediated deubiquitylation of TβRII promotes tumor progression and immune suppression, thereby fostering a tumor‐permissive microenvironment in pancreatic cancer. Given its role in stabilizing oncogenic signaling components, USP8 represents a potential therapeutic target in PDAC (Cui et al. 2023; Yang et al. 2023). TSG101 (tumor susceptibility gene 101) is implicated in exosome biogenesis and intracellular trafficking, both of which are critically involved in intercellular communication within the tumor microenvironment. Dysregulation of TSG101 function has been associated with aberrant exosome secretion, which can facilitate the horizontal transfer of oncogenic factors, microRNAs, and drug resistance mechanisms among pancreatic cancer cells. The accumulation of TSG101‐mediated exosomal cargo has been reported to enhance tumor invasiveness, immune evasion, and chemoresistance in pancreatic cancer (Ferraiuolo et al. 2020). Notably, exosomes harbor different potassium channels (Sanghvi et al. 2025).

In addition to the above known interactors, we identified a number of unexpected proteins in the K_Ca_3.1 proxisome with documented relevance for cancer development. Several of the newly identified interacting proteins, including ABCC5, have gained notoriety for their role in drug resistance and tumor cell survival under conditions of therapeutic stress. ABCC5 is a transporter protein that facilitates the efflux of chemotherapeutic drugs, thereby reducing intracellular drug accumulation and efficacy. The presence of other observed K_Ca_3.1 interactions, such as altered tumor suppressor proteins, including APC, SCRIB, WWOX, PTPN13, and PTPN14, underscores how changes in K_Ca_3.1 expression may trigger the loss of key regulatory mechanisms that normally control cell proliferation, adhesion, and polarity. APC, a well‐known tumor suppressor, is a key component of the Wnt/β‐catenin signaling pathway, which is frequently dysregulated in pancreatic cancer, leading to aberrant cell growth and survival (Song et al. 2024). SCRIB, a regulator of cell polarity, is often lost or mislocalised in pancreatic tumors, contributing to the transition from an epithelial to a more invasive mesenchymal phenotype. The loss of SCRIB drives the progression of invasive pancreatic cancer through both cell‐autonomous and non‐cell‐autonomous mechanisms, correlating with worse patient outcomes. This highlights SCRIB as a tumor suppressor and a potential biomarker for predicting recurrence (Bermejo‐Rodriguez et al. 2024). WWOX, the tumor suppressor WW domain‐containing oxidoreductase (WWOX), one of the most active fragile sites in the human genome, is commonly altered in pancreatic cancer. Loss of tumor suppressor WWOX accelerates pancreatic cancer development through promotion of TGFβ/BMP2 signaling (Husanie et al. 2022). PTPN13 and PTPN14, protein tyrosine phosphatases, modulate key signaling pathways involved in tumor progression and metastasis (Bollu et al. 2017; Tang et al. 2022).

### 4.4 K_Ca_3.1‐Related Signaling Network in Pancreatic Cancer

Signalling pathways associated with oncogenesis are also significantly represented among the identified proteins. ERBB2 (HER2) and MET are both well‐established oncogenes that drive tumor growth, survival, and therapy resistance (Arteaga and Engelman 2014; Hsu and Hung 2016; Mo and Liu 2017; Mo et al. 2022; Rubin et al. 2024). ERBB2, a receptor tyrosine kinase, is overexpressed in a subset of pancreatic cancers and a predictive tool for survival in patients with pancreatic cancer in histological studies (Ortega et al. 2022). ERBIN, a scaffold protein that modulates ERBB2 signalling, may further contribute to pancreatic cancer progression. Among the identified interactors, LRP6, a coreceptor in the Wnt signalling pathway, CTNNB1 (β‐catenin), and CTNND1 (p120‐catenin) represent pivotal components of Wnt and cadherin‐mediated adhesion signaling, both of which are frequently dysregulated in pancreatic cancer, thereby contributing to augmented cell proliferation and metastatic potential (Aguilera and Dawson 2021; Xue et al. 2024).

In addition, GO enrichment analysis also provides substantial evidence supporting K_Ca_3.1 involvement in multiple cellular processes, particularly those associated with cytoskeletal dynamics, signal transduction, and cell adhesion. The enrichment of actin filament organization and depolymerization processes suggests that K_Ca_3.1 interactors regulate cytoskeletal remodeling, which is essential for cell motility, migration, and invasion. This finding is consistent with previous research that K_Ca_3.1 contributes to tumor progression by modulating actin dynamics and cell shape changes. Furthermore, the significant enrichment of small GTPase‐mediated signalling pathways suggests a potential role for K_Ca_3.1 in modulating intracellular signal transduction cascades. Small GTPases, including Ras and Rho family members, are well known for their roles in proliferation, differentiation, and migration. The interaction of K_Ca_3.1 with GTPase signaling components suggests that it may act as a regulator of these pathways, influencing tumor progression and metastasis. The presence of membrane‐associated terms, including phosphatidylinositol binding, phospholipid binding, and membrane raft localization, indicates a possible involvement in lipid‐mediated signaling. Given that K_Ca_3.1 channels are known to be regulated by membrane lipids, this finding provides further mechanistic insights into their functional regulation and downstream signaling effects. A particularly intriguing finding is the strong enrichment of cell adhesion‐related terms, including cadherin binding, tight junctions, and adherens junctions. The association of K_Ca_3.1 with cell junctions and adhesion molecules suggests a role in maintaining cellular connectivity and tissue integrity, which may be disrupted in cancer progression. The observed interactions with cadherins and actin‐binding proteins further support this hypothesis, highlighting potential mechanisms by which K_Ca_3.1 may influence EMT and tumor dissemination.

By conducting a KEGG pathway analysis, we were able to identify multiple cellular processes and signalling pathways associated with potassium channel activity. These included Hippo signalling, focal adhesion, actin cytoskeleton regulation, calcium signalling, and endocytosis. The findings of this analysis suggest that ion homeostasis and ion transport mechanisms play a critical role in tumor growth, invasion, and therapy resistance in PDAC. The involvement of potassium channels in calcium signalling highlights an essential interplay in tumor cell survival and proliferation, particularly through the K_Ca_3.1, which has been reported to contribute to tumor progression by modulating intracellular Ca^2+^ levels, apoptosis, and cell motility. Our pathway analysis further confirms the association between ion transport and oncogenic signaling cascades, including MAPK, PI3K‐Akt, and Rap1, which drive tumor cell proliferation and survival.

A salient clinical finding from our analysis pertains to the potential involvement of potassium transport in drug resistance, as the enrichment of endocytosis‐related pathways, encompassing SNARE interactions in vesicular transport, clathrin‐mediated endocytosis, and FcγR‐mediated phagocytosis, suggests a contribution of potassium channels to chemoresistance by regulating drug uptake, intracellular trafficking, and lysosomal degradation. This finding is consistent with previous reports demonstrating that potassium channels influence vesicular transport and chemotherapy response. The Rap1 signaling pathway, which was also identified in our analysis, plays a crucial role in cell migration, angiogenesis, and tumor invasion, all of which are critical in PDAC progression. Furthermore, the adherens junction and tight junction pathways regulate cell adhesion and metastasis, and their disruption promotes pancreatic cancer invasiveness. Recent studies suggest that bacterial invasion of epithelial cells may impact PDAC tumorigenesis by altering the tumor microenvironment, while endocytosis contributes to membrane receptor regulation and therapeutic resistance mechanisms.

The activation or inhibition of K_Ca_3.1 channels has the capacity to modulate these pathways directly, given the evidence that, for example, Rap1 signalling governs migration and cell adhesion. Similarly, Adherens and Tight junction pathways are subject to potassium‐mediated modulation, affecting their dynamics and stability and ultimately facilitating or restricting tumor dissemination. Furthermore, K_Ca_3.1 channels have been shown to regulate membrane trafficking and receptor signalling in endocytosis, thereby altering PDAC survival and chemoresistance mechanisms. The strong association between potassium channels and oncogenic pathways suggests that targeting potassium transport represents a promising therapeutic approach in PDAC. Inhibition of K_Ca_3.1 channels has been shown to reduce tumor proliferation and enhance chemosensitivity in preclinical models (Liu et al. 2015; Chen et al. 2023), and our pathway analysis suggests that disrupting potassium‐dependent signalling cascades, such as Wnt/β‐catenin and MAPK signalling, may further potentiate therapeutic efficacy.

### 4.5 K_Ca_3.1 and Pancreatic Cancer—Going Beyond the Proxisome

The re‐evaluation of bioinformatics data after a 3‐year period highlights the dynamic nature of cancer genomics research, emphasizing the necessity for regular reassessment to maintain scientific accuracy, uncover novel biological insights, and ensure clinical relevance. The results of this study provide convincing evidence for the role of K_Ca_3.1 in PAAD and establish its potential as a prognostic biomarker.

A significant finding is the association between K_Ca_3.1expression and tumor stage, with the most pronounced elevation observed in Stage 4 tumor. This suggests that K_Ca_3.1 expression levels may be linked to tumor aggressiveness. However, the observation that metastatic samples exhibit expression levels comparable to or slightly lower than primary tumor suggests that K_Ca_3.1 may play a more significant role in early tumor progression rather than direct metastatic dissemination. This hypothesis requires further validation through functional studies to determine whether K_Ca_3.1 actively contributes to metastatic potential or primarily influences tumor growth and survival.

The correlation between K_Ca_3.1 expression and *TP53* mutations introduces an additional layer of complexity to its role in PAAD, suggesting a potential functional link between *TP53* loss‐of‐function and K_Ca_3.1 activation. *TP53*, a well‐established tumor suppressor, is frequently inactivated in tumor, and this is associated with enhanced tumour aggressiveness and resistance to therapy. The observed association suggests the possibility that K_Ca_3.1 may contribute to the aggressive phenotype of *TP53*‐mutant tumor, either by influencing cellular proliferation, survival, or adaptation to metabolic stress. Future studies should investigate the mechanistic interplay between *TP53* inactivation and K_Ca_3.1 upregulation, as this may uncover novel therapeutic vulnerabilities in PAAD.

Another salient aspect of this study pertains to the ethnicity‐based variation in K_Ca_3.1 expression, wherein a significantly higher upregulation was observed in Asian patients when compared to the absence of substantial differences in Caucasian and African American groups. This finding suggests the potential for ethnicity‐specific variations in K_Ca_3.1 regulation. However, the limited sample size of non‐Caucasian groups has been identified as a limitation, which hinders the study's statistical power. This necessitates further validation in larger, more diverse cohorts. If these findings are confirmed, they could have implications for the field of personalized medicine, as ethnicity‐related differences in K_Ca_3.1 expression might influence tumor behavior and response to treatment. The survival analysis further reinforces the potential clinical relevance of K_Ca_3.1 in PAAD.

The Kaplan–Meier curve and multivariate Cox regression analysis both indicate that high K_Ca_3.1 expression is associated with poor prognosis, independent of tumor stage, and the hazard ratio of 1.31 suggests a 31% increased risk of mortality in patients with elevated K_Ca_3.1 levels, establishing its prognostic value. Furthermore, the observed association between elevated age and unfavorable prognosis underscores the necessity for a multifaceted approach that incorporates both molecular and clinical factors in the stratification of patients with PAAD for risk assessment and treatment planning.

Although we defined the interactome of K_Ca_3.1 specifically in a PDAC cell line, the documented expression of K_Ca_3.1 in the tumor‐infiltrating immune cells (Panyi et al. 2014) may also have relevance in the context of PDAC (Hofschröer et al. 2021). Cell‐type specific differences in K_Ca_3.1 interactome are likely to occur, but many interactors, such as, for example, STIM1/2, may be conserved. In this respect, the present study indicates an association between potassium channel activity and leukocyte trans endothelial migration, thus suggesting a potential role in immune cell infiltration and immune evasion. The collective findings from immune cell profiling, genetic variations, and pathway analyses highlight K_Ca_3.1 as a key modulator of immune responses in PAAD, reinforcing its association with an immunosuppressive tumor microenvironment. The immune cell heatmap revealed a positive correlation between K_Ca_3.1 and immunosuppressive cell types, such as Tregs and macrophages, and a negative correlation with cytotoxic and effector immune cells (CD8^+^ T cells, NK cells, gamma delta T cells). These findings indicate K_Ca_3.1's role in promoting immune evasion and tumor progression. This finding was further validated by SNV (single‐nucleotide variants)‐based immune infiltration analysis, which demonstrated that mutant groups exhibited higher macrophage and Treg abundance but reduced CD8^+^ and NK cell infiltration, mirroring the immune landscape associated with high expression. Furthermore, CNV (copy number variations) analysis confirmed that gene amplification is linked to a decrease in immune‐activating cells (CD4^+^ T cells, gamma delta T cells) and an enrichment of immunosuppressive Th2 cells, suggesting that genetic alterations in K_Ca_3.1‐related pathways further shape an immune‐resistant microenvironment. The association between GSVA scores and oncogenic pathway activity provided additional support, showing that Tregs, neutrophils, and monocytes—previously linked to *KCNN4* —positively correlate with active cancer pathways, while cytotoxic and effector immune cells are suppressed. These observations emphasize the role of *KCNN4* in coordinating immune resistance mechanisms, suppressing antitumor immunity, and facilitating tumor progression.

In conclusion, the present findings indicate that *KCNN4* could be considered a viable therapeutic target in PAAD, given that its inhibition has the potential to restore immune infiltration, counteract immune evasion, and enhance the effectiveness of immunotherapy. It is recommended that future research efforts concentrate on ascertaining whether *KCNN4*‐directed therapies can effectively modulate the immune microenvironment, with the objective of enhancing immune activation and tumor control in pancreatic cancer. Altogether, these findings prompt the hypothesis that potassium channel inhibitors could be utilized in combination with immunotherapy to enhance antitumor immune responses in PDAC, representing a novel therapeutic approach with the potential to improve patient outcomes.

## 5 Conclusion

This study provides novel insights into the intricate network of interactions involving K_Ca_3.1 in pancreatic cancer, emphasizing its strong association with oncogenic pathways and reinforcing its potential as a therapeutic target. The findings highlight the multifaceted role of K_Ca_3.1 in PAAD, encompassing tumor progression, immune modulation, and mitochondrial dynamics. Further functional validation is necessary to clarify the mechanistic interplay between K_Ca_3.1 and its interacting partners, particularly in pathways related to tumor invasion, immune evasion, and drug resistance, and to nail down the specific role of mitochondrial K_Ca_3.1 in cancer‐related processes.

The identification of K_Ca_3.1 both as a prognostic biomarker and a promising therapeutic target carries significant implications for immunotherapy and precision medicine. Future research should focus on evaluating the therapeutic potential of potassium channel inhibitors, particularly in combination strategies, to enhance antitumor immune responses and ultimately improve clinical outcomes for pancreatic cancer patients.

## Author Contributions

Conceptualization: Vanessa Checchetto, Ildikò Szabò, and Veronica Carpanese. Methodology: Vanessa Checchetto, Ildikò Szabò, Veronica Carpanese, Soha Sadeghi, and Luca Matteo Todesca. Investigation: Veronica Carpanese, Soha Sadeghi, and Luca Matteo Todesca. Writing – original draft: Vanessa Checchetto, Ildikò Szabò, Veronica Carpanese, Soha Sadeghi, and Luca Matteo Todesca. Editing: Veronica Carpanese, Ildikò Szabò, Vanessa Checchetto, Soha Sadeghi, and Luca Matteo Todesca. Funding: Vanessa Checchetto and Ildikò Szabò. Supervision: Vanessa Checchetto and Ildikò Szabò. All authors read, edited, and approved the final manuscript.

## Conflicts of Interest

The authors declare no conflicts of interest.

## Supporting information

Supp information.

Table 1 Comprehensive list of proteins identified through the BioID analysis.

## Data Availability

The data that support the findings of this study are available from the corresponding author upon reasonable request.
