# Supplementary material for: Intermediate Conductance Calcium‐Dependent Potassium Channel (KCa3.1) Interacting Proteins Using Turboid‐Based Proximity Labeling Technology: Insights Into Interactome and Related Signaling Pathways in Pancreatic Tumors
Source: J Cell Physiol. 2025 Sep 15;240(9):e70092. doi: 10.1002/jcp.70092 (PMC12435150; doi:10.1002/jcp.70092)
Supplement: Supplementary file 1 — Supp information. [file JCP-240-0-s002.pdf]

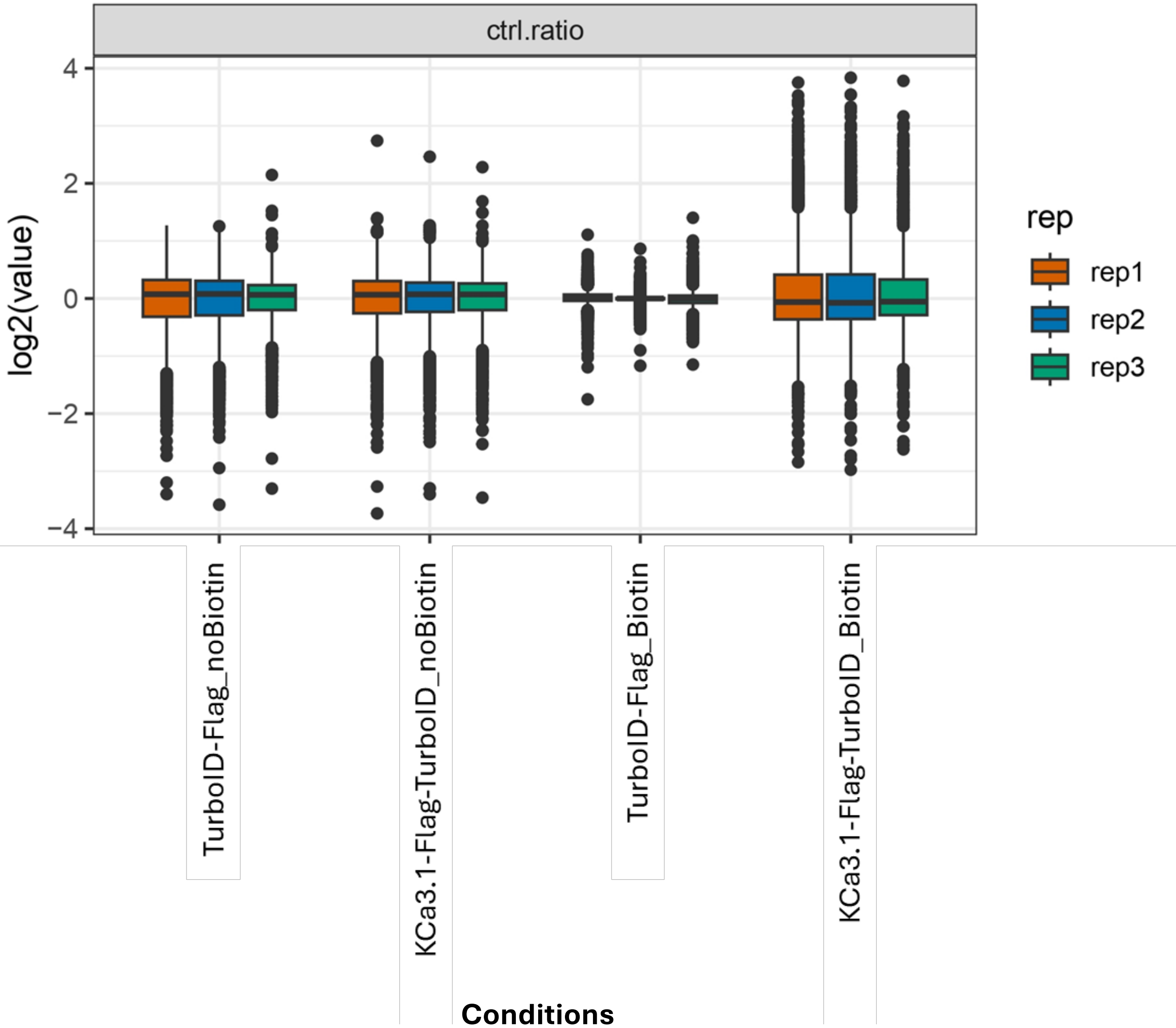

ctrl.ratio
4
2
0
−2
−4
log2(value)
rep
rep1
rep2
rep3
TurboID-Flag_noBiotin
KCa3.1-Flag-TurboID_noBiotin
TurboID-Flag_Biotin
KCa3.1-Flag-TurboID_Biotin
Conditions

Figure S1

Figure S1: **Box plot of log2(value) distribution across experimental conditions.** Box plot showing the distribution of log2 values for different experimental conditions. Colors represent different biological replicates (*rep1*, *rep2*, and *rep3*). The y-axis displays $\log_2$ values, while the x-axis represents conditions. The data distributions suggest similar variation among replicates for each condition, with greater dispersion observed in biotinylated samples.

Figure S2

Figure S2: **Heatmap of Gene Expression Across Experimental Conditions.** (**A**) Heatmap displaying the log_2 ratio of gene expression across different experimental conditions. The color scale represents expression changes, where red indicates higher expression levels (log_2 ratio > 0) and blue represents lower expression levels. The biotinylated conditions show a marked increase in expression for specific genes compared to the no-biotin conditions.

A

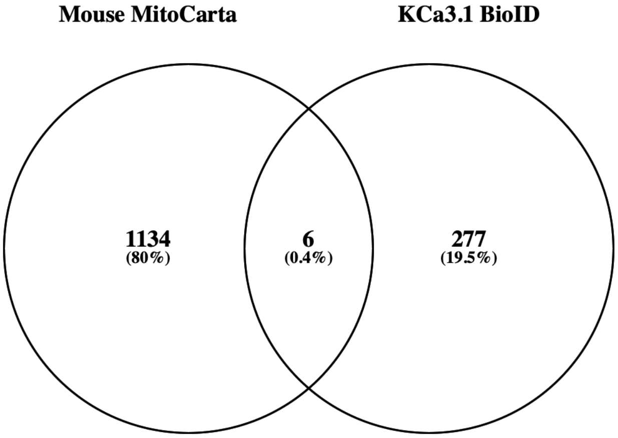

Figure S3

Figure S3: Overlap between the mitochondrial proteome and K_{Ca}3.1 interactors (**A**) Venn diagram showing the overlap between proteins listed in the Mouse MitoCarta database and those identified as KCa3.1 interactors through BioID. 6 proteins (0.4%) are shared between the two datasets, suggesting a limited but specific mitochondrial association of KCa3.1 interactors.

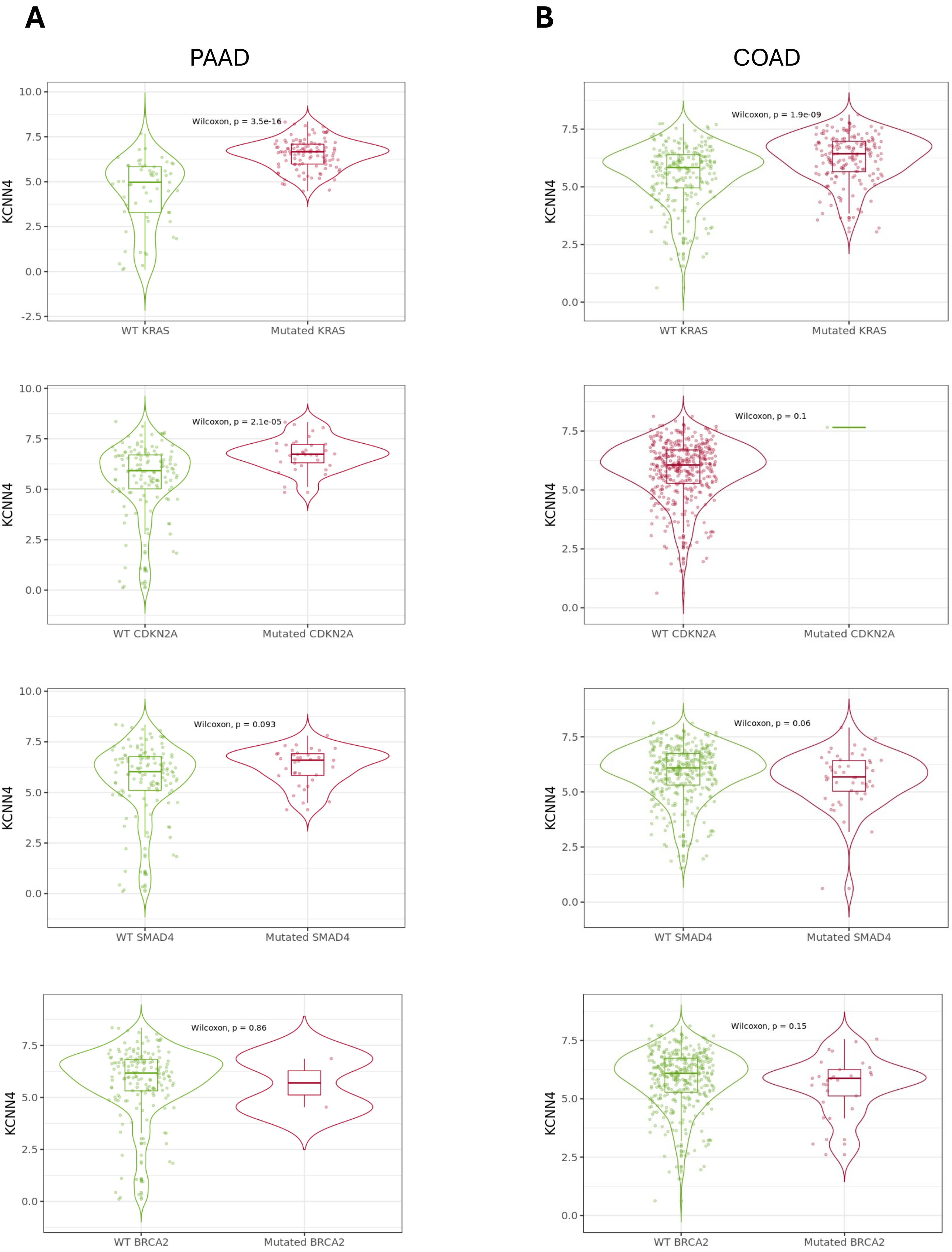
A
B
PAADAutomatic
COAD
Wilcoxon, p = 3.5e-16
Wilcoxon, p = 1.9e-09
Wilcoxon, p = 2.1e-05
Wilcoxon, p = 0.1
Wilcoxon, p = 0.093
Wilcoxon, p = 0.06
Wilcoxon, p = 0.86
Wilcoxon, p = 0.15
KCNN4
WT KRAS
Mutated KRAS
WT CDKN2A
Mutated CDKN2A
WT SMAD4
Mutated SMAD4
WT BRCA2
Mutated BRCA2

Figure S4

Figure S4: KCNN4 expression in relation to common Driver mutations in PAAD and COAD. Violin plots showing the expression of KCNN4 4 in tumor samples from PAAD (left panels) and COAD (right panels), stratified by mutation status of key cancer driver genes: KRAS, CDKN2A, SMAD4, and BRCA2. In PAAD, KCNN4 is significantly upregulated in tumors with mutated KRAS (p = 3.5e-16) and CDKN2A (p = 2.1e-05), while no significant association is observed for SMAD4 (p = 0.093) or BRCA2 (p = 0.86). In COAD, KRAS mutations are also associated with increased KCNMA4 expression (p = 1.3e-09), whereas mutations in CDKN2A (p = 0.1), SMAD4 (p = 0.06), and BRCA2 (p = 0.15) do not show significant differences. Statistical comparisons were performed using the Wilcoxon rank-sum test.

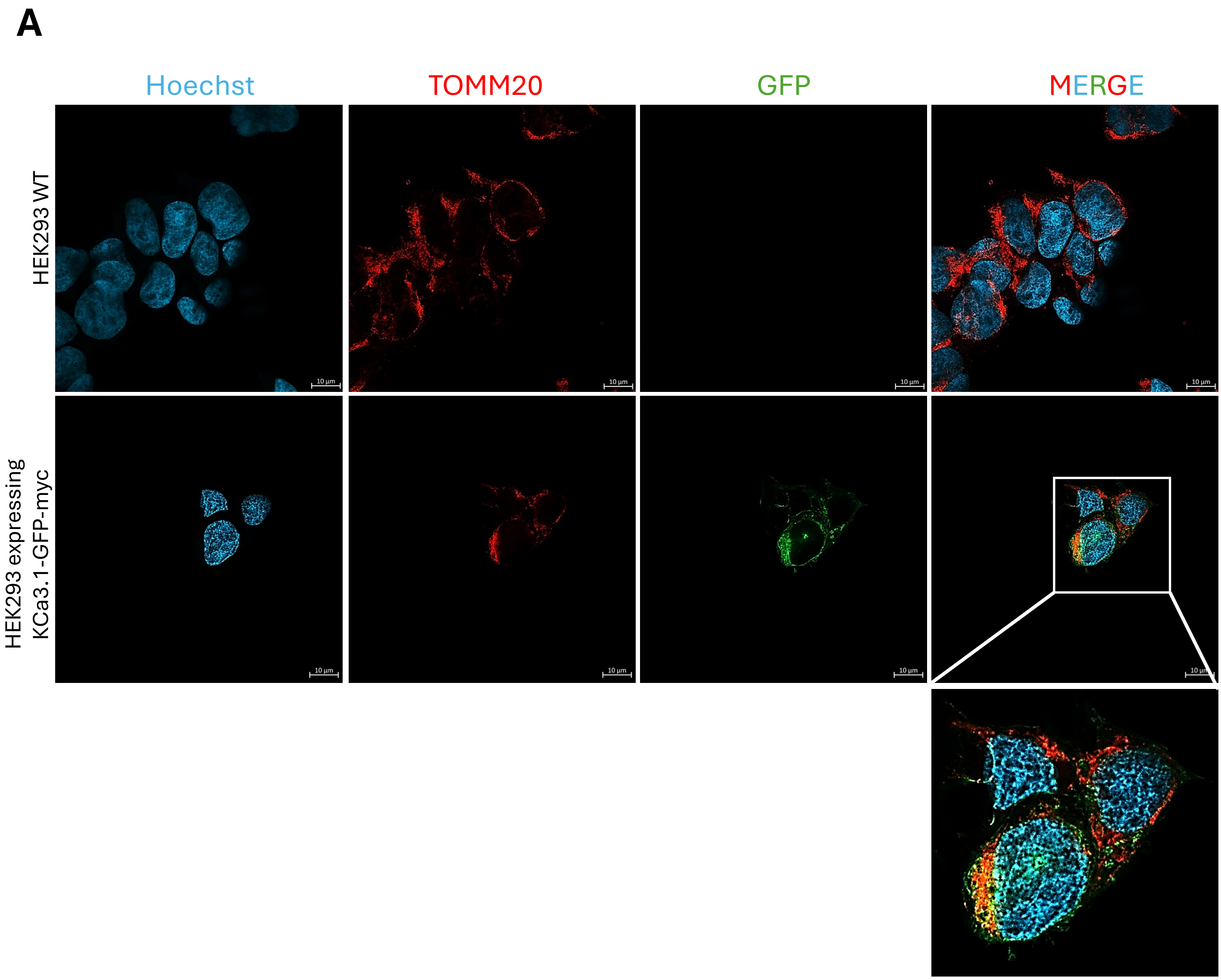

Figure S5 continued

B

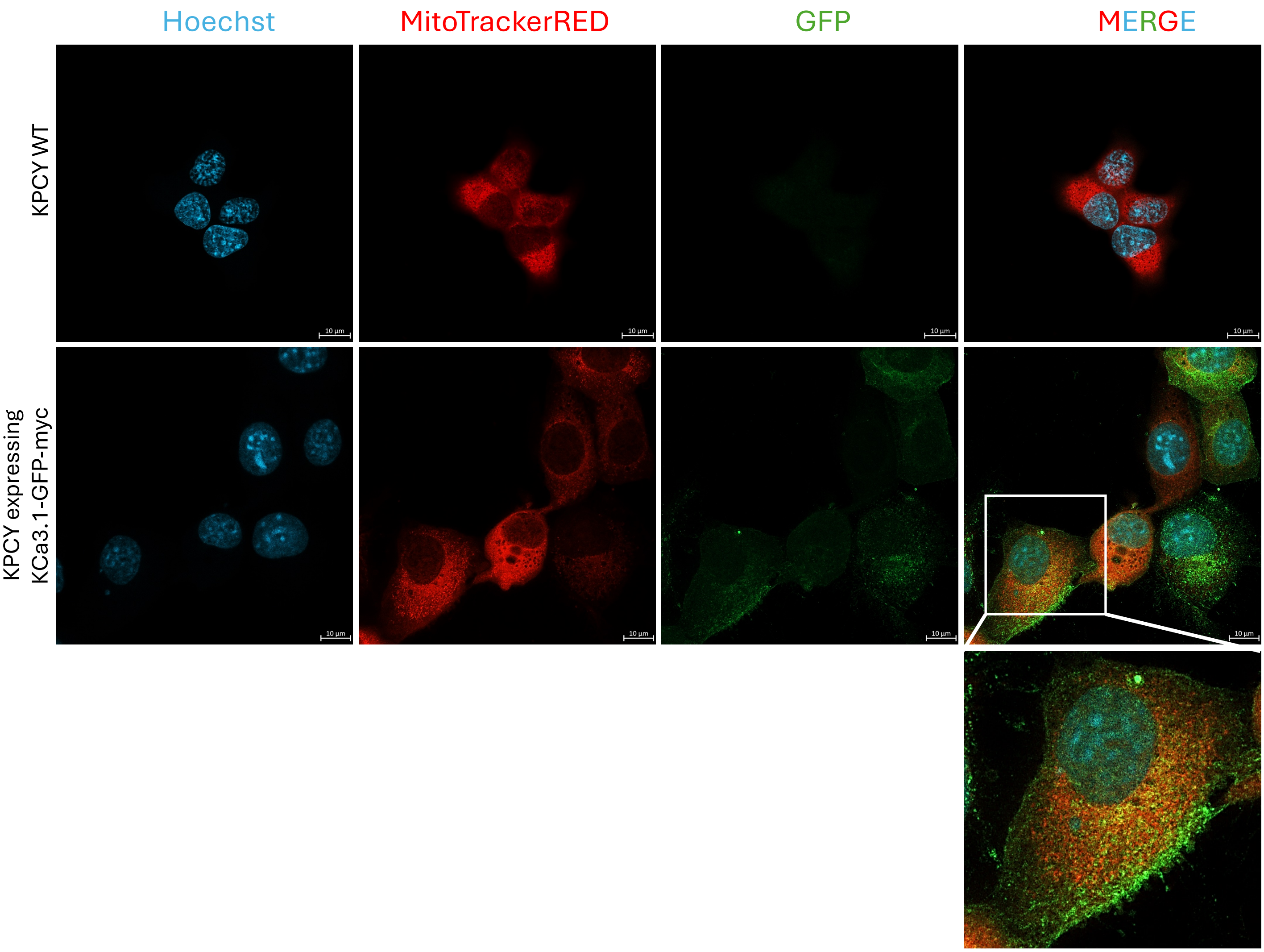

Figure S5 continued

C

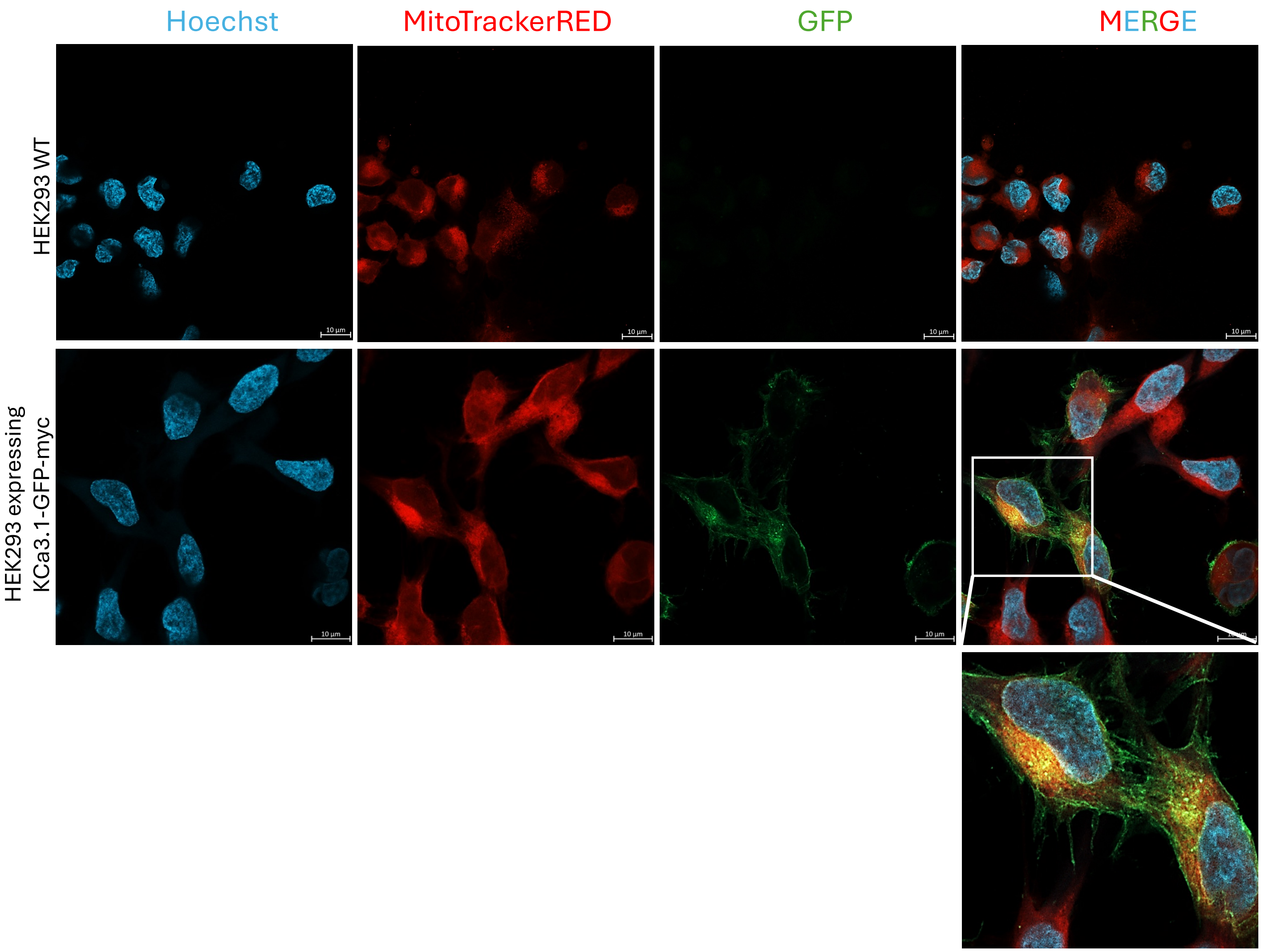
Hoechst
MitoTrackerRED
GFP
MERGE
HEK293 WT
HEK293 expressing
KCa3.1-GFP-myc
10 µm

Figure S5

Figure S5: Confocal microscopy analysis of K_{Ca}3.1-GFP-myc subcellular localization in KPCY and HEK293 cells. (**A**): HEK293 wild-type (WT) and KCa3.1-GFP-myc-overexpressing cells were immunostained with an anti-TOMM20 antibody (red) to visualize mitochondria. KCa3.1 is visualized in green via the GFP tag. Nuclei were counterstained with Hoechst (blue). (**B-C**): MitoTracker Red CMXRos staining of KPCY and HEK293 WT and KCa3.1-GFP-myc-overexpressing cells to label mitochondria. KCa3.1 is shown in green (GFP), and nuclei are stained with Hoechst (blue).

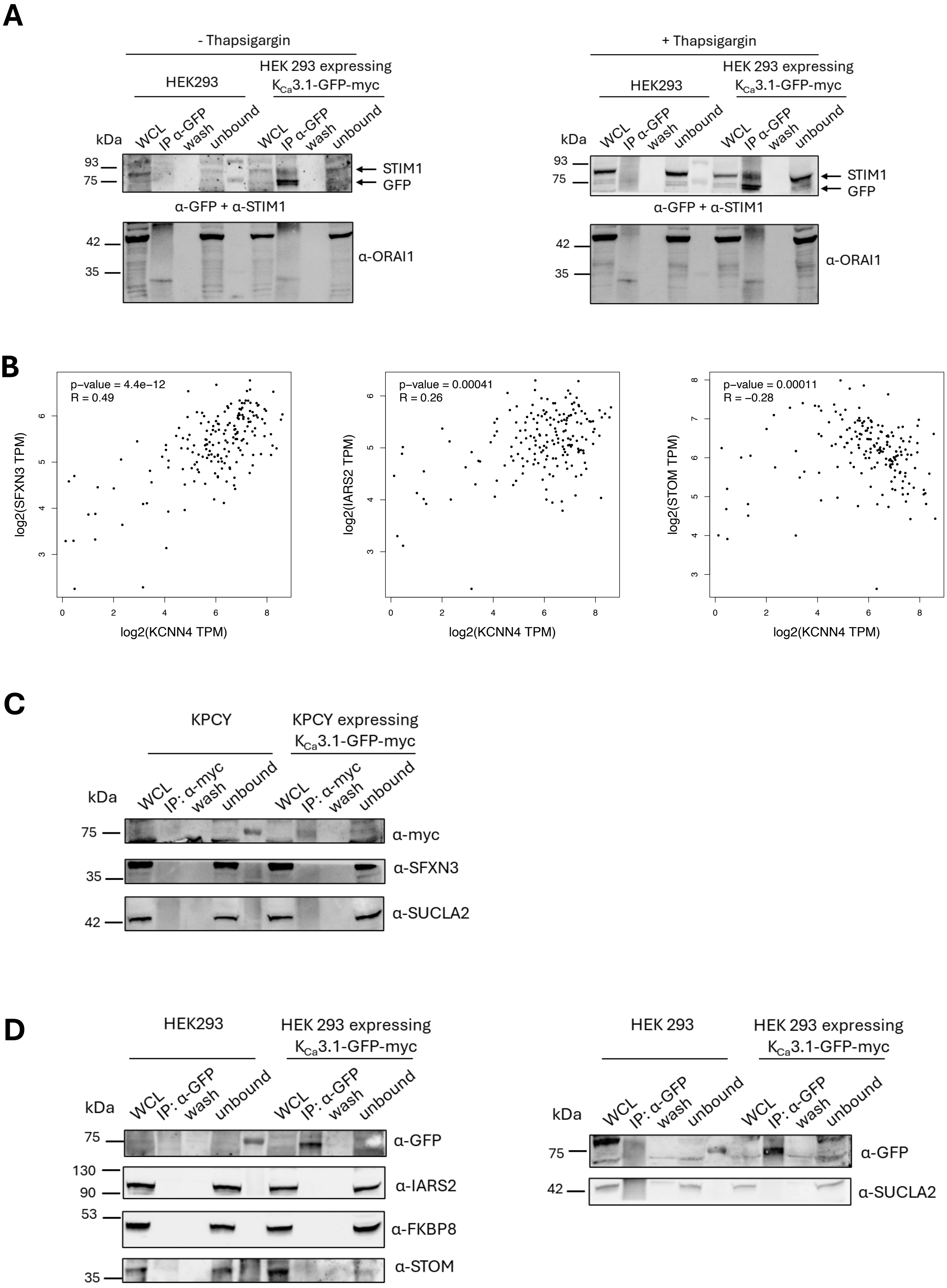
A
- Thapsigargin
HEK293
HEK 293 expressing
K$_{Ca}$3.1-GFP-myc
kDa
WCL
IP α-GFP
wash
unbound
93
75
STIM1
GFP
α-GFP + α-STIM1
42
35
α-ORAI1
+ Thapsigargin
B
p–value = 4.4e−12
R = 0.49
log2(SFXN3 TPM)
log2(KCNN4 TPM)
p–value = 0.00041
R = 0.26
log2(IARS2 TPM)
p–value = 0.00011
R = −0.28
log2(STOM TPM)
C
KPCY
KPCY expressing
K$_{Ca}$3.1-GFP-myc
IP: α-myc
α-myc
α-SFXN3
α-SUCLA2
D
IP: α-GFP
α-GFP
130
90
α-IARS2
53
α-FKBP8
α-STOM
HEK 293

Figure S6

Figure S6: Immunoprecipitation of K_{Ca}3.1 with partners identified by interactomic analysis. (**A**) K_{Ca}3.1 immunoprecipitates with STIM1 under both basal and SOCE-activated conditions. Despite the observed interaction between K_{Ca}3.1 and STIM1, no detectable interaction with Orai1 was observed under the same conditions. (**B**) Correlation between *KCNN4* and Mitochondrial Genes in PAAD Tumor. Scatter plots display the correlation between KCNN4 expression and the indicated mitochondrial genes in PAAD tumor based on TCGA and GTEx datasets. Pearson's correlation coefficient (R) and p-values are indicated in each plot. (**C**) Immunoprecipitation of K_{Ca}3.1 with mitochondrial partners. Immunoprecipitation was performed using an anti-myc antibody to pull down myc-GFP-tagged K_{Ca}3.1 in KPCY cells. Immunoblot analysis of the precipitates using α-SFXN3 and α-SUCLA2 antibodies revealed no detectable physical interaction between K_{Ca}3.1 and these mitochondrial proteins. (**D**) Immunoprecipitation was performed using an anti-GFP antibody to pull down myc-GFP-tagged K_{Ca}3.1 in HEK293 cells. in KPCY cells. Immunoblot analysis of the precipitates using α-IARS2, α-FKBP8, α-STOM and α-SUCLA2 antibodies revealed no detectable physical interaction between K_{Ca}3.1 and these mitochondrial proteins.
